# A radiomics-based model for predicting prognosis of locally advanced gastric cancer in the preoperative setting

**DOI:** 10.1038/s41598-021-81408-z

**Published:** 2021-01-21

**Authors:** Jaeseung Shin, Joon Seok Lim, Yong-Min Huh, Jie-Hyun Kim, Woo Jin Hyung, Jae-Joon Chung, Kyunghwa Han, Sungwon Kim

**Affiliations:** 1 Department of Radiology and Research Institute of Radiological Science, Severance Hospital, Yonsei University College of Medicine, 50-1 Yonsei-ro, Seodaemun-gu, Seoul, 03722 South Korea; 2 Department of Internal Medicine, Gangnam Severance Hospital, Yonsei University College of Medicine, Seoul, South Korea; 3 Department of Surgery, Yonsei University College of Medicine, Seoul, South Korea; 4 Department of Radiology, Gangnam Severance Hospital, Yonsei University College of Medicine, Seoul, South Korea

**Keywords:** Gastrointestinal cancer, Prognostic markers, Cancer imaging

## Abstract

This study aims to evaluate the performance of a radiomic signature-based model for predicting recurrence-free survival (RFS) of locally advanced gastric cancer (LAGC) using preoperative contrast-enhanced CT. This retrospective study included a training cohort (349 patients) and an external validation cohort (61 patients) who underwent curative resection for LAGC in 2010 without neoadjuvant therapies. Available preoperative clinical factors, including conventional CT staging and endoscopic data, and 438 radiomic features from the preoperative CT were obtained. To predict RFS, a radiomic model was developed using penalized Cox regression with the least absolute shrinkage and selection operator with ten-fold cross-validation. Internal and external validations were performed using a bootstrapping method. With the final 410 patients (58.2 ± 13.0 years-old; 268 female), the radiomic model consisted of seven selected features. In both of the internal and the external validation, the integrated area under the receiver operating characteristic curve values of both the radiomic model (0.714, *P* < 0.001 [internal validation]; 0.652, *P* = 0.010 [external validation]) and the merged model (0.719, *P* < 0.001; 0.651, *P* = 0.014) were significantly higher than those of the clinical model (0.616; 0.594). The radiomics-based model on preoperative CT images may improve RFS prediction and high-risk stratification in the preoperative setting of LAGC.

## Introduction

Gastric cancer is the fourth most common cancer and third leading cause of cancer-related deaths worldwide^1,2^. Complete R0 resection with subsequent adjuvant chemotherapy is effective on patients with locally advanced gastric cancer (LAGC)^3^. However, recurrence after complete resection occurs in up to 30–40% of patients within 5 years^4–6^. Recently, neoadjuvant chemotherapy is widely recommended in international western guidelines for advanced gastric cancer patients because of its potential benefits, including early treatment of micrometastases, delivery of higher dose chemotherapy before surgery, and an improved down-staging change of the primary tumor^3^. Higher R0 resection rate and survival can be achieved with neoadjuvant chemotherapy followed by curative surgery^7,8^. As evidence supporting neoadjuvant chemotherapy accumulates, identification of patients as neoadjuvant candidates becomes important.

CT is the modality of choice for preoperative clinical staging of gastric cancer; however, studies have reported limitations regarding staging accuracy and risk stratification^9^. Due to intrinsic limitations of CT spatial resolution in distinguishing gastric wall layers, tumor staging is suboptimal. Preoperative CT-based node staging is also limited because size-based differentiation of small lymph nodes (LNs) with micrometastasis from large reactive LNs is difficult^10^. Hence, there is a growing need to use biomarkers in conjunction with abdominal CT to predict the prognosis of LAGC.

Radiomics has emerged as a promising tool for discovering new imaging biomarkers by converting digital medical images into high-dimensional quantitative features^11–13^. Its potential capacity to capture useful information and increase diagnostic and prognostic power has been demonstrated in lung, prostate, brain, liver, and colorectal cancers^14^. Although several studies^15–17^, they were limited by the small sample size and lack of validation. Recently, a large retrospective study^18^ demonstrated that the radiomics signature had good performance in predicting prognosis and survival benefit of adjuvant chemotherapy. However, the study included a considerable number of gastric cancer cases with early stage or distant metastasis, which limited the risk stratification of patients with LAGCs who are subject to preoperative chemotherapy.

This study aimed to develop and validate a radiomics-based prognostic model for recurrence-free survival (RFS) using preoperative contrast-enhanced CT in LAGC. Moreover, we assessed the value added by radiomic signatures when integrated with clinical profiles in the preoperative setting and whether the radiomics model can perform risk stratification for tumor recurrence.

## Results

The total radiomics quality score^11^ was 15 (adherence rate 15/36, 41.7%) in 16 domains (Supplementary Table S1).

### Study population characteristics

**Figure 1:**
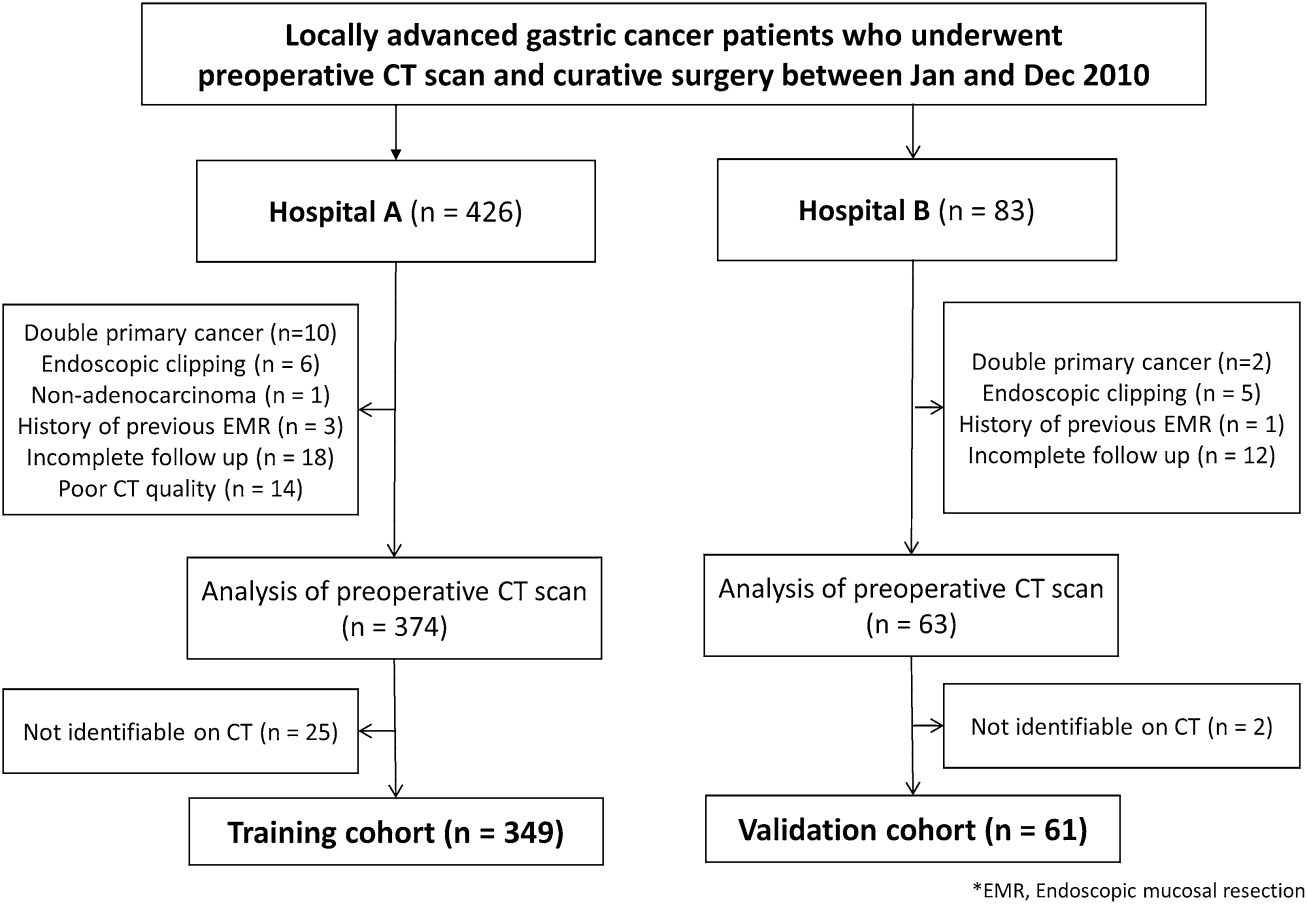
Flowchart for patient selection in training cohort and validation cohort.

A total of 410 patients (mean age, 58.2 ± 13.0 years; 268 men) was included in the final study population, with 349 patients (mean age, 58.3 ± 12.6 years; 232 men) in the training cohort and 61 patients (mean age, 57.3 ± 15.0 years; 40 men) in the validation cohort (Fig. 1). There was no significant difference between training and validation cohorts in recurrence, sex, age, carcinoembryonic antigen, carbohydrate antigen 19-9, T stage, N stage, differentiation, Lauren classification, and lymphovascular invasion (Table 1). In the training cohort, recurrences occurred in 95 of 349 patients (27.2%) and the 1-, 2-, and 5-year cumulative global RFS rates were 92.2%, 85.7%, and 75.1% (95% confidence interval (CI) [89.4, 95.1], [82.1, 89.5], [70.6, 80.0]), respectively. In the validation cohort, recurrences occurred in 21 of 61 patients (33.3%) and the 1-, 2-, and 5-year cumulative global RFS rates were 86.0%, 76.5, and 64.1% (95% CI [77.5, 95.5], [66.1, 88.6], [52.2, 78.6]), respectively. Nodular extramural infiltration in CT showed significant difference between the two cohorts (Table 1).

**Table 1:** Patient characteristics in the training and validation cohorts.

	Training (n = 349)	Validation (n = 61)	*p* value
Recurrence (%)	95 (27.2)	21 (33.3)	0.249
Sex (female, %)	117 (33.5)	25 (41.0)	0.259
Age (mean ± SD)	58.3 ± 12.6	57.3 ± 15.0	0.601
CEA (elevated, %)	33 (9.5)	4 (6.6)	0.466
CA 19–9 (elevated, %)	35 (10.0)	9 (13.8)	0.271
**Conventional CT features**			
Size (mean ± SD)	47.3 ± 24.9	50.0 ± 19.8	0.347
Tumor depth : Nodular extramural (n, %)	138 (39.5)	12 (19.7)	0.003
cN2 – 3 (n, %)	80 (22.9)	9 (14.8)	0.153
Borrmann type 4 (n, %)	31 (8.9)	8 (13.1)	0.299
**Endoscopy data**			
Differentiation (n, %)			0.801
Well/moderate	126 (36.1)	21(34.4)	
Poorly/undifferentiated	223 (63.9)	40 (65.6)	
Location (n, %)			0.127
Upper	57 (16.3)	14 (23.0)	
Middle	103 (29.5)	10 (16.4)	
Lower	180 (51.6)	34 (55.7)	
Whole	9 (2.6)	3 (4.9)	
Borrmann type 4 (n, %)	11 (3.1)	4 (6.6)	0.191
**Surgical pathology data**			
T stage (n, %)			0.607
T2	94 (26.9)	10 (16.4)	
T3	113 (32.4)	21 (34.4)	
T4a	140 (40.1)	30 (49.2)	
T4b	2 (0.6)	0 (0.0)	
N stage (n, %)			0.785
N0	126 (32.6)	20 (32.7)	
N1	70 (22.0)	10 (16.4)	
N2	70 (20.1)	12 (19.7)	
N3a	55 (16.5)	12 (19.7)	
N3b	28 (8.8)	7 (11.5)	
Differentiation (n, %)			0.066
Well/moderate	122 (35.0)	14 (23.0)	
Poorly/undifferentiated	227 (65.0)	47 (77.0)	
Lauren classification (n, %)			0.119
Intestinal	175 (50.1)	24 (39.3)	
Diffuse/mixed	174 (49.8)	37 (60.7)	
LV invasion (positive, %)	154 (44.1)	26 (42.6)	0.827
Borrmann type 4 (n, %)	25 (7.2)	7 (11.5)	0.246

SD, standard deviation; CEA, carcinoembryonic antigen; CA, carbohydrate antigen; LV invasion, lympho-vascular invasion.

In the final study population of 410 patients, R0 gastrectomy with D2 lymphadenectomy was successfully performed with 140 (34.1%) total gastrectomy and 270 (64.1%) subtotal gastrectomy. TNM stage III patients represented 49.5% (203/410) while TNM stage II and I represented 36.8% (151/410) and 14.4% (59/410), respectively. Overall, 74.1% of the patients received adjuvant chemotherapy (stage III, 94.6%; stage II, 68.2%; stage I, 15.3%).

### Feature selection and radiomics signature building

The interobserver and interslice intraclass correlation coefficient (ICC) ranges were 0.491–1.000, and 0.360–0.965, respectively. Therefore, 240 features with ICC > 0.75 on both interobserver and interslice reproducibility were used for the further analysis.

In the least absolute shrinkage and selection operator (LASSO) Cox regression model, a value of tuning parameter lambda (λ) = 0.077 with log (λ) = − 2.58 was selected by ten-fold cross-validation to minimize partial likelihood deviance values among 240 features. The optimal tuning parameter resulted in seven non-zero coefficients (Supplementary Fig. S1). The radscore was calculated as follows:

$$ \begin{aligned} {\text{Radscore }} & = \, ( - {8}.{6617}0{5} \times {\text{original}}\_{\text{shape}}\_{\text{Sphericity}}) \\ & \quad + \, ( - {1}.{974956} \times {\text{original}}\_{\text{glcm}}\_{\text{Imc1}}) \\ & \quad + \, ({1}.0{33112} \times {\text{original}}\_{\text{glcm}}\_{\text{Imc2}}) \\ & \quad + \, ( - 0.{517325} \times {\text{original}}\_{\text{glszm}}\_{\text{SmallAreaEmphasis}}) \\ & \quad + \, ({1}.{67}0{9}0{1} \times {\text{wavelet}}.{\text{LH}}\_{\text{glcm}}\_{\text{Idmn}}) \\ & \quad + \, ({5}.{198918} \times {1}0^{{ - {5}}} \times {\text{wavelet}}.{\text{LH}}\_{\text{gldm}}\_{\text{GrayLevelNonUniformity}}) \\ & \quad + \, ( - {9}.{272848} \times {1}0^{{ - {9}}} \times {\text{wavelet}}.{\text{HL}}\_{\text{glszm}}\_{\text{LargeAreaHighGrayLevelEmphasis}}). \\ \end{aligned} $$

### Model construction and radscore performance evaluation

Performance of the clinical, radiomics, and merged models were evaluated. Among preoperative clinical factors, Tumor depth on CT (CT-Depth) and Tumors classified as Borrmann type 4 on CT (CT-Type 4) were identified as independent factors for predicting RFS using backward stepwise approach (Table 2).

**Table 2:** Preoperative clinical factors for predicting tumor recurrence-free survival.

Clinical feature		Univariate analysis		Multivariate analysis*	
		HR (95% CI)	*p* value	HR (95% CI)	*p* value
Age	≤ 60	Reference			
	> 60	1.326 (0.875–2.012)	0.184		
Sex	Male	Reference			
	Female	1.189 (0.773–1.828)	0.431		
CEA	< 5 U/ml	Reference			
	≥ 5 U/ml	1.557 (0.828–2.927)	0.169		
CA 19–9	< 37 U/ml	Reference			
	≥ 37 U/ml	2.143 (1.189–3.863)	0.011†		
CT-Size	≤ 4 cm	Reference			
	> 4 cm	2.469 (1.513–4.030)	< 0.001†		
CT-Depth	Nodular extramural infiltration ( −)	Reference		Reference	
	Nodular extramural infiltration ( +)	2.103 (1.385–3.194)	< 0.001†	1.899 (1.237–2.915)	0.003†
CT-LN status	cN0 or cN1	Reference			
	cN2 or cN3	1.696 (1.079–2.666)	0.022†		
CT-Borrmann type	Type 1,2, or 3	Reference		Reference	
	Type 4	2.646 (1.539–4.549)	< 0.001†	2.174 (1.247–3.789)	0.006†
Endoscopy-Location	Upper	Reference			
	Middle	2.059 (0.938–4.518)	0.072		
	Lower	2.057 (0.973–4.349)	0.059		
	Whole	5.155 (1.686–15.764)	0.004†		
Endoscopy-Histological grade	Well or moderate	Reference			
	Poorly differentiated	1.328 (0.841–2.097)	0.224		
Endoscopy-Borrmann type	Type 1,2, or 3	Reference			
	Type 4	1.884 (0.764–4.645)	0.169		

HR, hazard ratio; CI, confidence interval; CEA, carcinoembryonic antigen; CA, carbohydrate antigen.

*The multivariate regression model was built using backward stepwise approach with Akaike information criteria.

^†^ Statistically significant.

**Figure 2:**
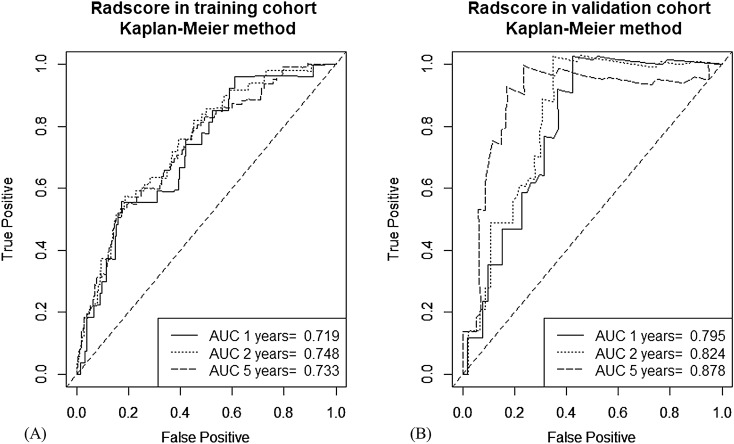
Survival receiver operating characteristic curves at 1, 2, and 5 years with the radscore, (**A**) in training cohort and (**B**) in validation cohort. R software (version 3.3.2, https://www.r-project.org) was used to draw.

The radscore prognostic accuracy on time-dependent receiver operating characteristic curves as measured by area under the curves at 1, 2, and 5 years were 0.719, 0.748, and 0.733 in training and 0.795, 0.824, and 0.878, in validation cohorts, respectively (Fig. 2).

The integrated area under the receiver operating characteristic curves (iAUC) values for RFS prediction in the internal validation were: 0.616, 95% CI [0.570, 0.663] in clinical; 0.714, 95% CI [0.667, 0.759] in radiomic; and 0.719, 95% CI [0.674, 0.764] in merged models. In external validation, the iAUC values were: 0.584, 95% CI [0.544, 0.636] in clinical; 0.652, 95% CI [0.628, 0.674] in radiomic; and 0.651, 95% CI [0. 630, 0.673] in merged models, respectively (Table 3).

**Table 3:** Model performances measured by iAUC for prediction of recurrence-free survival.

Model	Internal validation			External validation		
	iAUC (95% CI)	iAUC Difference*	*p* value	iAUC (95% CI)	iAUC Difference*	*p* value
Clinical	0.616 (0.570, 0.663)	–	–	0.594 (0.544, 0.636)	–	–
Radiomic	0.714 (0.667, 0.759)	0.098	< 0.001†	0.652 (0.628, 0.674)	0.056	0.010†
Clinico-radiomic	0.719 (0.674, 0.764)	0.102	< 0.001†	0.651 (0.630, 0.673)	0.057	0.014†

iAUC, the integrated area under the receiver operating characteristic curve; CI, confidence interval.

*Comparison with clinical model.

^†^Statistically significant.

The radiomic model showed higher iAUC values than the clinical model in both internal (iAUC difference = 0.098, *p* < 0.001) and external validations (iAUC difference = 0.056, *p* = 0.010). Similarly, the merged model showed higher iAUC values than the clinical model in both internal (iAUC difference = 0.102, *p* < 0.001) and external validations (iAUC difference = 0.057, *p* = 0.014) (Table 3).

### Radscore-based risk stratification

**Figure 3:**
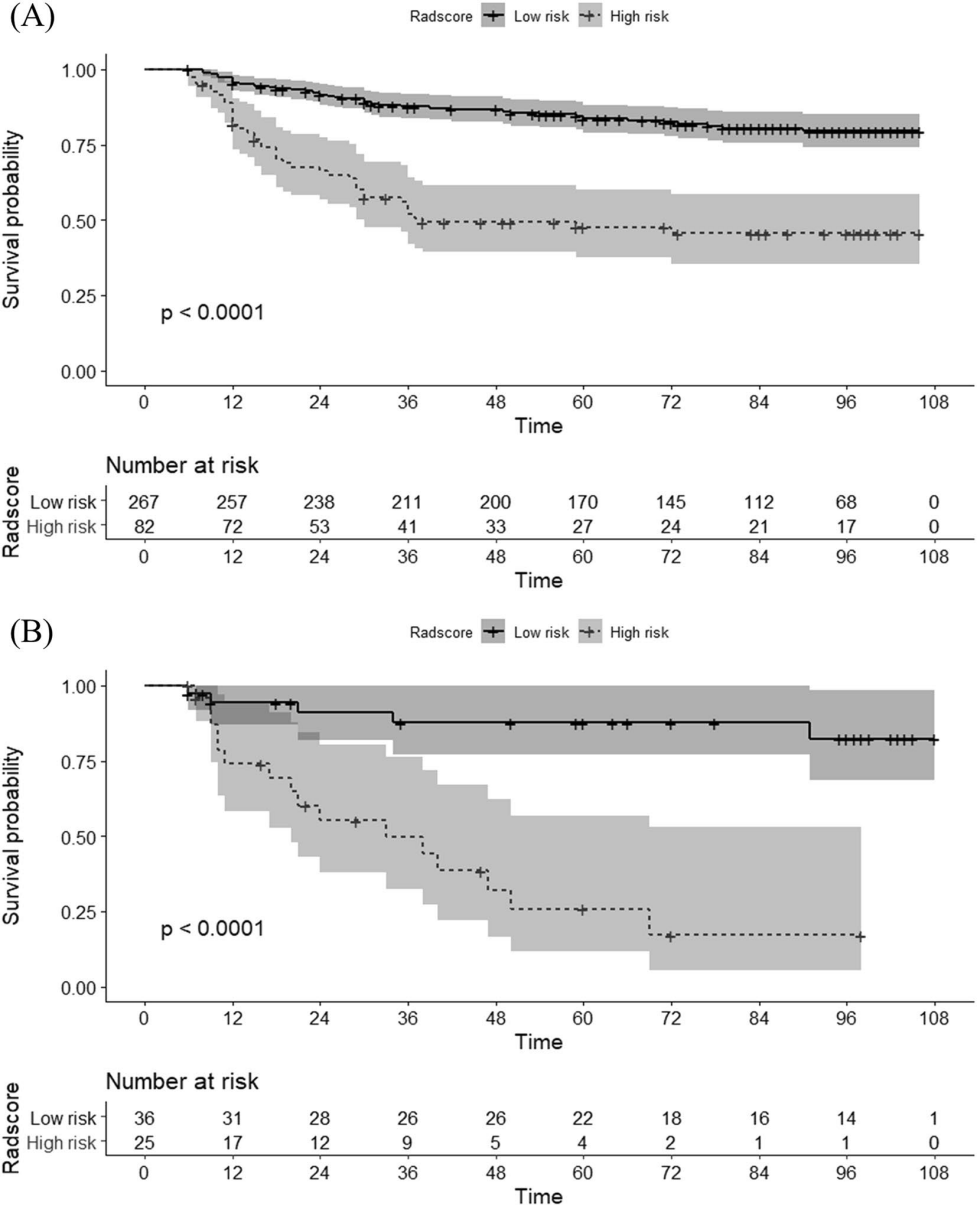
Kaplan–Meier curves and risk tables for recurrence-free survival (RFS) from (**A**) the training (n = 349) and (**B**) validation (n = 61) cohorts. Patients were stratified on the basis of the cutoff (radscore = 1.164) to maximize log-rank statistic. The radiomics score significantly stratified the patients into low- and high-risk groups for RFS in the training cohort (*p* < 0.001; log-rank test) and the validation cohort (*p* < 0.001; log-rank test). Shaded areas represent 95% confidence intervals. R software (version 3.3.2, https://www.r-project.org) was used to draw.

**Figure 4:**
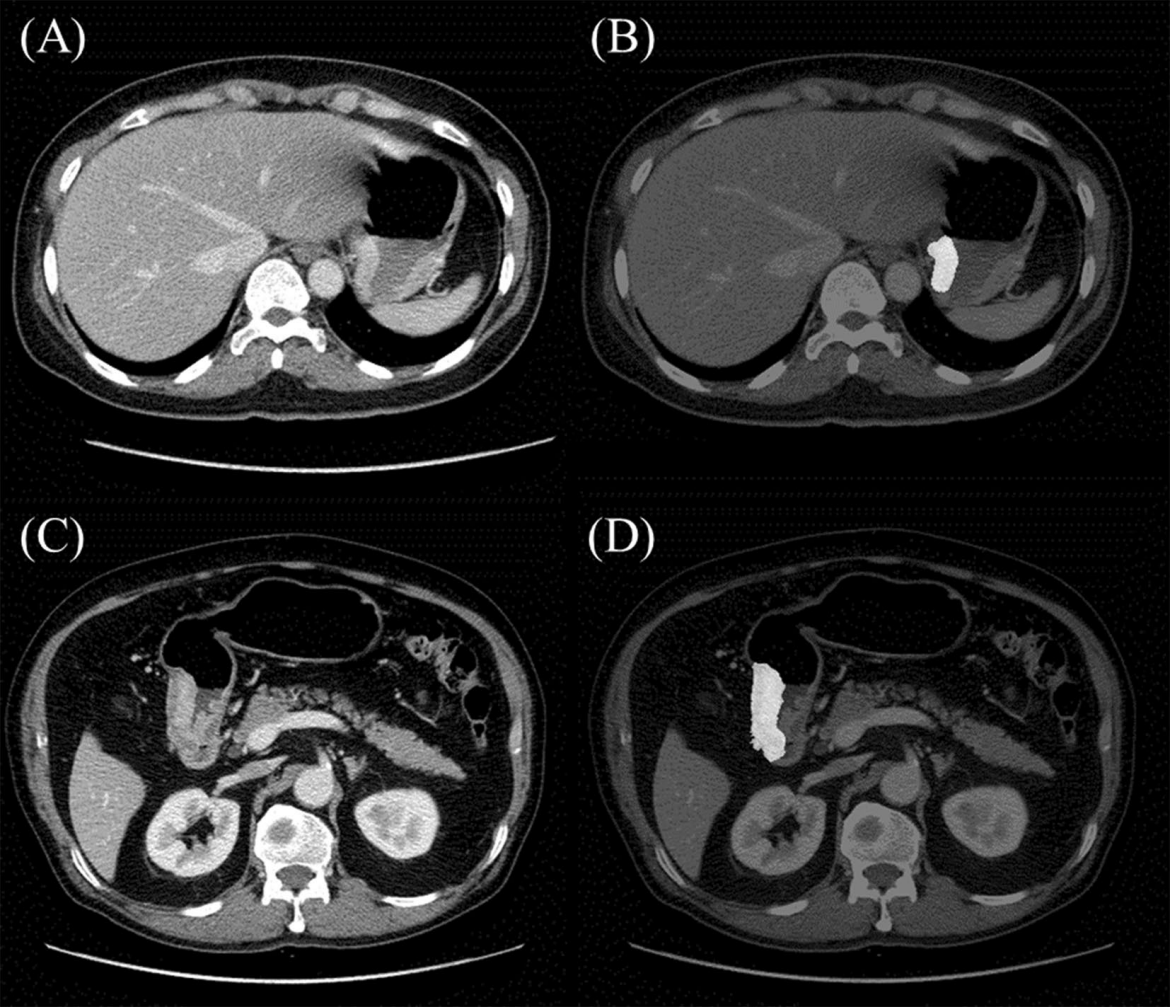
Patients with locally advanced gastric cancer whose recurrence risk was stratified into high and low risk by the radscore cutoff 1.16. (**A**) Computed tomography (CT) images on portal venous phase, (**B**) tumor segmentation in a 52-year-old woman with nodular extramural infiltration on CT whose radscore was 0.98, low risk group. Preoperative carcinoembryonic antigen (CEA) and carbohydrate antigen (CA) 19–9 was within the normal limit. Surgical pathology revealed tumor-node-metastasis (TNM) stage IIb with T4a and N0. There was no tumor recurrence during 96 months after surgery. (**C**) CT images on portal venous phase, (**D**) tumor segmentation in a 67-year-old man without nodular extramural infiltration on CT whose radscore was 1.66, high risk group. Preoperative CEA and CA 19–9 was within the normal limit. Surgical pathology revealed TNM stage IIIc with T4a and N3a. Liver metastasis occurred at 12 months after surgery.

The patients were classified into low- and high-risk groups based on radscore cutoffs (1.116) selected from the training set using maximally selected log-rank statistics. In both training and validation cohorts, high-risk patients showed significantly lower RFS than low-risk patients. RFS hazard ratios, hazard ratios were 4.209 (95%CI [2.787, 6.357], *p* < 0.001) and 22.061 (95%CI [5.571, 87.36], *p* < 0.001) in training and validation cohorts, respectively (Fig. 3). Examples of patients with high and low risk by the radscore are shown in Fig. 4.

**Figure 5:**
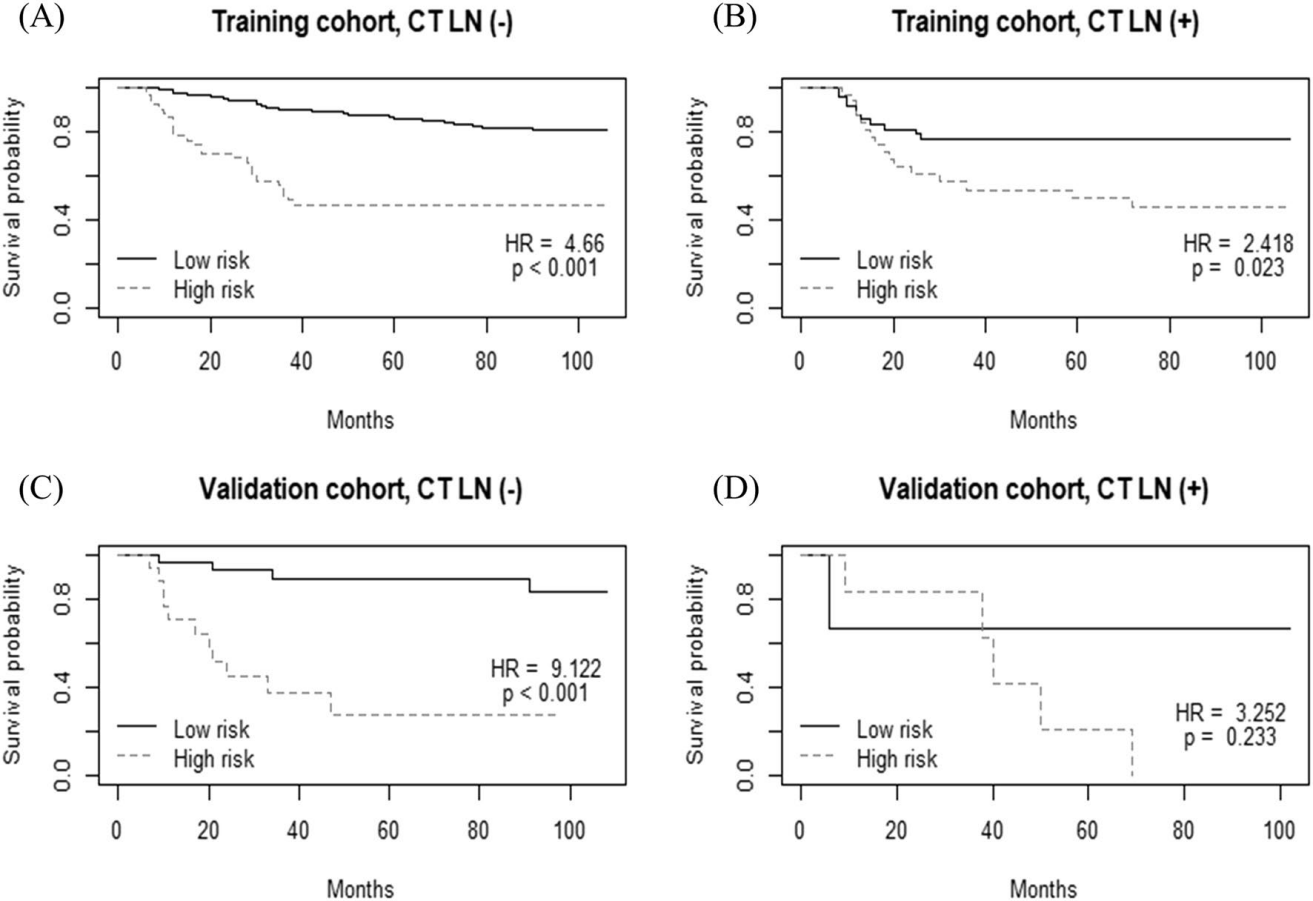
Kaplan–Meier survival analysis of recurrence-free survival according to the radiomics score classifier in subgroups of the training and validation cohorts. (**A**) Training cohort, lymph node (LN) stage 0 or 1 on CT (n = 269). (**B**) Training cohort, LN stage 2 or over on CT (n = 80). (**C**) Validation cohort, LN stage 0 or 1 on CT (n = 52). (**D**) Validation cohort, LN stage 2 or over on CT (n = 9). R software (version 3.3.2, https://www.r-project.org) was used to draw.

To assess the ability of radiomics to predict early recurrence, patients who underwent follow-up for more than two years were divided into two groups based on recurrence within two years. There was a significant difference in the radiomic scores between these two groups in both training (0.96 ± 0.50 vs. 0.56 ± 0.59; *p* < 0.001) and validation cohorts (1.20 ± 0.41 vs. 0.76 ± 0.33; *p* = 0.014). When patients were dichotomized according to CT-Size, CT-Depth, and CT-Type4, and adjuvant chemotherapy, the Kaplan–Meier curves of the high- and low-radscore groups showed a p value < 0.05 in the validation group (Figures S4–S7). However, the Kaplan–Meier curves for RFS of the high- and low-radscore groups were not significantly different in the CT-LN ( +) group of the validation cohort (*p* = 0.233) (Fig. 5).

## Discussion

To predict prognosis for RFS in patients with LAGC using preoperative CT, we identified the radscore consisting of seven radiomic features and verified its value through external validation. In preoperative setting, the radscore was an independent prognostic factor in both training and validation cohorts and showed good RFS predicting performance in LAGC and outperformed the clinical model alone. The merged model showed significantly higher prognostic performance than the clinical model, indicating that the radiomic model added value to the clinical model-based prediction. The results support the clinical application of radiomics in providing additional information for LAGC-treatment decision-making in the preoperative setting, without any additional invasive procedure. Moreover, the high performance of radiomic model on risk-stratification may help in selecting candidates for investigational treatments.

Even though pathologic TNM stage is still the most reliable prognostic factor for long-term outcomes of gastric cancer^19,20^, such data can only be obtained after the completion of surgery. Preoperative treatment could alter the pathologic stage, therefore, development of non-invasive biomarkers that provide guidance for adjusting the therapeutic approach is essential for LAGC. Several studies have recently highlighted the prognostic potential of texture analysis or radiomics in patients with gastric cancer^15,18,21^. A large-scale retrospective study demonstrated that radiomics signature has more prognostic value than clinicopathological features^18^. However, their study population included a considerable proportion of patients with early stage gastric cancer or distant metastatic stage. Early gastric cancer is known to have an excellent prognosis without needing chemotherapy and the AGC with distant metastasis is known to require systemic chemotherapy without resection surgery^22,23^. We targeted LAGC since a variety of treatments have been proposed but gray zones persist in treatment determination. Our study revealed that radiomics had higher prognostic performance than the clinical model, suggesting that radiomics could be a practical imaging biomarker for patients with LAGC in a preoperative setting. However, the merged model did not perform better than the radiomics model. This might be attributed to the possibility that most characteristics of the clinical model (mainly based on conventional imaging characteristics), were already reflected in the radscore.

The usefulness of neoadjuvant chemotherapy in LAGC is still controversial. Large-scale phase III trials in Europe have reported that perioperative chemotherapy has survival benefits over surgical treatment alone^24,25^. However, in the majority of cases in these studies, a proper lymphadenectomy was not performed during the surgery. Furthermore, a lack of information about initial tumor staging before treatment could lead to selection bias^10^. In Korea and Japan, D2 lymphadenectomy is generally performed along with gastrectomy in resectable advanced gastric cancer, therefore the usefulness of neoadjuvant chemotherapy has not been concluded yet^22,23^. This controversy could be resolved through risk stratification, i.e., by identifying gastric cancer cases with a high risk of recurrence. In our study, radscore was successfully dichotomized into high- and low-risk groups, and verified by external validation. Therefore, radscore could offer guidance for therapeutic strategies depending on recurrence risk, thereby improving the clinical outcome. Particularly, LAGC patients classified as high-risk based on the radscore may be ideal candidates for neoadjuvant treatment, given that its potential benefits outweigh the morbidity risk and higher treatment cost. However, since patients with neoadjuvant treatment were not included in this study, its efficacy could not be assessed using radscore. Further study is required to evaluate the correlation between radscore and response to neoadjuvant therapy.

We evaluated the characteristics and applicability of the radscore in various clinical conditions. The radscore showed successful risk stratification in each subgroup dichotomized according to tumor size, tumor depth, or Borrmann types on preoperative CT. This indicates that the radscore could provide a more sophisticated risk stratification independent of known clinical prognostic factors. The radscore might help predict prognosis of LAGC, regardless of the outcome of current preoperative clinical staging. Interestingly, the radscore could significantly distinguish patients into two risk groups, only in the clinically LN negative subgroup, but not in the clinically LN positive subgroup. However, the number of patients with LN stage 2 or over on CT in the validation cohort was too small (n = 9) to have statistical power. Moreover, clinical N staging by preoperative CT is very limited in LAGC patients^10^, even though relatively satisfactory sensitivity and specificity have been reported for ≥ pN2 stage^26^, which was used as the cutoff in our study. Although the targets of radiomics were limited to primary tumors, and since metastatic LN was not included in this radiomics analysis, it is needed further study to confirm the prognostic power of the radscore in the LN positive group.

Among the seven features in the radscore, the sphericity, which is selected from the shape features, quantifies the roundness of the shape of the tumor region relative to a circle. Any lesion with low sphericity could be associated with a flat or infiltrative tumor, which has been regarded as Borrmann type 4. Two gray level co-occurrence matrix (GLCM)-related features in the radscore, informational measure of correlation 1 (IMC1) and IMC2, measure the complexity of the texture patterns^27^. Selected Small-Area-Emphasis of gray level size zone matrix features (GLSZM) measures the distribution of small size zones, with a greater value indicative of smaller size zones and finer textures^27^. These GLCM and GLSZM features have specific mathematical formula measuring different aspects of textural heterogeneity within the tumor, e.g. tissue necrosis. These GLCM- or GLSZM-based texture features reflecting the interaction between neighboring pixels have shown better quantification of tumor texture and heterogeneity than histogram-based features^28^. In addition, three features from the wavelet decompositions of original images are also included in our radscore. By focusing on different frequency ranges within the tumor, features from wavelet decompositions might be able to reveal the characteristics of tumors that did not appear in the original image.

This study has several limitations. First, it was a retrospective study with a relatively small sample size; however, the number was similar to those in previous radiomic studies^29,30^. Moreover, external validation with cohort from the spatially separate hospital was performed to overcome this limitation. Future study with a larger sample for both training and validation is required for a robust prediction model. Second, the recurrence rate in the training cohort was 27.2%, imbalanced data. Any approach to rebalance the dataset was not performed to preserve representative of the clinical situation. In addition, in this study, LASSO Cox regression was performed to build the radscore, instead of machine learning technique. Third, the proportion of cases with nodular infiltration on CT was different between the training and validation cohorts, presumably due to different scale and clinical settings of the two spatially separate hospitals. Nevertheless, the radscore showed significant risk stratification in both cohorts. Fourth, since only patients who did not receive neoadjuvant chemotherapy were included, the benefits of neoadjuvant therapy in high versus low radscore groups could not be evaluated. Further study in a large prospective cohort, randomized by neoadjuvant chemotherapy status is needed to integrate this technology into clinical practice. Fifth, clustering for radiomics features to remove redundancy was not performed before model building and highly correlating features (Supplementary Fig. S2), such as IMC1 and IMC2, were included. These features were linear combined in the radscore and the effect of redundancy might be small. Sixth, images from different machines or manufacturers of CT were included in the training cohort and fourteen patients were excluded with poor quality of CT. To minimize variability from different CT scanners, we used a uniform acquisition protocol and resampled the images into the same pixel spacing. Moreover, the validation was performed on the cohort from a different hospital. Standardized protocol for different CT scanners is required for future study and application of radiomics prediction model in clinical setting. Seventh, feature extraction was performed from a single slice with the largest lesion, similar to the previous study^18^. Although tumor evaluation on a single CT section might not be representative of the entire tumor characteristics^31^, previous studies reported that two-dimensional features showed prognostic performance comparable with three-dimensional segmentations in non-small cell lung cancer and rectal cancer^32,33^. However, there is still controversy whether two-dimensional segmentation can replace recommended three-dimensional segmentation which allows comprehensive assessment of whole tumor. Lastly, the tumors were outlined semi-automatically, which may be time-consuming and user-dependent in terms of selecting the slice containing the largest area of the tumor and region of interest placement. To reduce variability in these processes, only features with excellent inter-slice and inter-reader ICCs were included for analysis. In future studies, automated 3D tumor segmentation based on deep learning would allow further automation of the workflow, minimize user bias, and enable larger studies.

In conclusion, radiomic signature based on preoperative CT images is a possible preoperative imaging biomarker that can improve RFS prediction of the preoperative clinical profile in LAGC. The ability of radiomic signatures to identify high-risk LAGC patients may be helpful in selecting appropriate candidates for neoadjuvant therapy.

## Methods

### Study population

This retrospective study was approved by the Institutional Review Board of Severance Hospital (Protocol no. 4-2019-0062) and the requirement to obtain written informed consent was waived. All methods described in this manuscript were performed in accordance with the approved guidelines and regulations.

From January 1, 2010 to December 31, 2010, consecutive patients with LAGC (pT2–4) underwent curative surgery without neoadjuvant therapy at a tertiary hospital, which is overlapped with the population of the previous study^34^. For external validation, consecutive patients from another tertiary hospital were collected with the same enrollment criteria. Patients were excluded if they had double primary cancer, histology other than adenocarcinoma, less than 6 months of follow-up, endoscopic clipping, and history of endoscopic mucosal resection. Patients with poor quality CT images, including slice thickness more than 5 mm or pixel size larger than 1.0 mm × 1.0 mm were also excluded. After CT image analysis, patients with no identifiable lesion on their CT scans were excluded. The final training and validation cohorts consisted of 349 and 61 patients, respectively (Fig. 1).

Clinical, laboratory, endoscopic, and pathological data were retrieved from patients’ electronic medical records, including serum levels of carcinoembryonic antigen, carbohydrate antigen 19–9, tumor location and size, differentiation, lymphovascular invasion, Lauren type, and tumor-node-metastasis (TNM) stage. The TNM staging was reclassified according to the eighth edition of the American Joint Committee on Cancer/ Union for International Cancer Control staging system. The following clinical factors were integrated in the preoperative clinical model: Age (≤ 60 vs. > 60 years); Sex (male vs. female); levels of serum carcinoembryonic antigen (< 5 vs. ≥ 5 U/ml) and carbohydrate antigen 19–9 (< 37 vs. ≥ 37 U/ml); endoscopy result including tumor location (upper vs. middle vs. lower), histological grade from biopsy tissue (well or moderate vs. poorly differentiated), Borrmann type (type 4 vs. others) (Table 2).

After surgical resection, all patients were followed up at our institution for 6.5 to 109.2 months (median follow-up: 71.5 months) through December 2018 according to our institutional protocol^35^. The RFS was defined from the date of surgery to recurrence at any site (event) or the last follow-up date (censored).

### CT image acquisition

CT scans were performed with a 16- or 64-channel multidetector CT scanner (Somatom Sensation 16 and Sensation 64; Siemens Medical Solutions, Forchhein, Germany; and Lightspeed VCT, GE Healthcare, Milwaukee, WI, USA). Images were acquired from the diaphragm level to the symphysis pubis with detector collimations of 16 × 0.75 mm (Somatom Sensation 16, Simens Medical Solutions), 64 × 0.6 mm (Somatom Sensation 64, Simens Medical Solutions), or 64 × 0.625 mm (Lightspeed VCT, GE healthcare). Other scanning parameters were as follows: tube current 160 mAs (Somatom Sensation 16 and Sensation 64, Siemens Medical Solutions) and 100–300 mAs of Automated tube current modulation with a noise index of 15 (AutomA; Lightspeed VCT, GE Healthcare); tube voltage 120 kVp; table speed, 24 mm per rotation; and gantry rotation time, 0.5 s. The details regarding the acquisition parameters of CT image are presented in Supplementary materials (Supplementary Table S2). For gastric distention, either gas distention with two packs of effervescent granules or water distention with 1 L of water was introduced. Scanning was performed during portal phases, as determined with bolus tracking and automated triggering technique after intravenous administration of 120–150 mL of nonionic contrast materials (300mgI/mL) using an automatic injector at a rate of 4 ml/second. The amount of contrast medium per patient was determined by the total body weight. Axial and coronal images were reconstructed with 3-mm-thick sections and a 3 mm interval with filtered back projection algorithm. From the Picture Archiving and Communication System (Centricity, GE Medical Systems, Milwaukee, WI, USA), portal venous phase CT images were retrieved for qualitative image review and radiomic feature extraction because the tumor tissue was well differentiated from the adjacent normal gastric tissue.

### CT image analysis

Preoperative CT images were independently reviewed by two board-certified abdominal radiologists with more than 10 years of subspecialty experience, who arrived at a consensus in cases with discrepancy. The CT imaging characteristics analyzed were tumor depth, LN status, tumor size, and Borrmann type. CT-Depth was categorized into nodular or less than nodular extramural infiltration groups—one of the major discriminating factors for predicting recurrence of AGC in a previous study^34^. LN involvement on CT (CT-LN) was categorized into two groups, N0–1 and N2–3, as multidetector CT might be useful for selecting candidates for neoadjuvant therapy with ≥ pN2 disease^26^. LNs were considered metastatic if they had a short-axis diameter > 8 mm. Tumor size (CT-Size) was measured as the longest diameter on the axial or coronal plane. Tumors were classified as CT-Type4 when infiltrative stomach cancer showed no definite ulceration or mass formation on preoperative CT^34^.

### Radiomics feature extraction

A 4th year radiology resident (J.S.) selected one axial image among the CT images that depicted the largest area of the lesion, under the inspection of an abdominal radiologist (J.S.L., 16-year experience). The CT images were resampled by pixel spacing 1.0 mm × 1.0 mm using the BSpline interpolator of Insight Segmentation and Registration Toolkit (ITK) package (https://www.itk.org). A free-form region of interest (ROI) was drawn along the margins of the tumor using semi-automatic methods aided by the CT attenuation threshold, measured using an open-source application, Medical Image Processing, Analysis, and Visualization (MIPAV) (https://mipav.cit.nih.gov). Each selected image and ROI were thoroughly checked by another abdominal radiologist (J.S.L.) with 16-year subspecialty experience. Disagreements about the ROI were resolved by consensus-based discussion. The radiologists were blinded to the clinical and histopathologic data, except for information on the diagnosis of gastric cancer and the general location of the tumor (upper, middle, lower, or whole) based on findings of the preoperative endoscopy, since we were not evaluating the detection ability.

Pyradiomics (version 2.0.0), the open-source python package, was used to extract radiomics features, including shape-based features, first-order features, and texture features (Supplementary Table S3). The original CT image was decomposed into four decompositions (low–high, high-high, high-low, low-low subbands) using two-dimensional coiflet wavelets, and radiomic features except for shape features were extracted from the wavelet transformed images. Finally, 438 tumor imaging quantifying features were obtained (94 features from original image and 86*4 features from the wavelet transformed images). Detailed information in radiomics feature extraction process can be found in Supplementary materials.

### Inter-observer and inter-slice agreement for selected features

Another board-certified abdominal radiologist (S.K.) with 5 years of subspecialty experience drew ROIs in 30 randomly selected lesions to analyze inter-observer reproducibility. The radiologist was blinded to the clinical and histopathological data except for the general location of the tumor. Inter-observer agreement was evaluated by the ICC based on a two-way random effect model. As only one slice with the largest section of the lesion was selected to draw ROI, inter-slice agreement among extracted features was calculated using ICC with 30 randomly chosen images of three consecutive slices including the largest section in the middle. The features with both inter-observer and inter-slice ICC greater than 0.75, which were suggested to be categorized into good to excellent reproducibility^27^, were included in subsequent analyses.

### Feature selection and radiomics signature building

The LASSO Cox regression^36^ was used to select the most prognostically useful texture features. Then, a multiple-feature based radiomics signature, namely the radscore, was constructed for predicting survival in the training cohort. Ten-fold cross-validation in the training set was performed to optimize hyperparameters for model generalizability.

### Model construction and radscore performance assessment

All available clinical factors in the preoperative setting were included in the clinical model building (Supplementary materials). In the training cohort, the clinical model for predicting RFS was built using the multivariable Cox proportional hazards model with a backward stepwise approach based on the Akaike information criteria. The radiomic model was built using radscore in a univariate Cox model. The radscore was incorporated into preoperative clinical model to build a merged clinico-radiomic model to evaluate the potential value of the radscore. The performances of the three models were evaluated in the external validation cohort using the iAUCs^37^ with 1000 bootstrap resamples.

### Radscore-based risk stratification

The potential association of radscore with RFS was assessed in training and validation cohorts using Kaplan–Meier survival analysis. The patients were stratified into high- and low-radscore groups, using a maximally selected log-rank statistic-based threshold^38^. The threshold value determined in the training cohort was applied to the validation cohort. Differences in survival distributions between the two groups were compared using log-rank tests. Subgroup analyses according to CT-Size, CT-LN, CT-Depth, CT-Type4, and adjuvant chemotherapy were performed to determine if there was any survival difference between the high- and low-radscore groups.

### Statistical analyses

Continuous variables were described using mean ± standard deviation and/or median with interquartile range and compared using independent t-tests. Categorical variables were compared using chi-squared or Fisher’s exact tests. RFS was assessed by the Kaplan–Meier method, and differences in survival distributions between groups were compared using log-rank tests. The multivariate Cox proportional hazards model with backward stepwise approach was used to identify independent clinical prognostic factors for RFS. Outcomes were expressed as hazard ratios and 95% CIs. To quantify the discrimination performance, iAUC values and their differences between models were calculated using a bootstrapping method (resampled 1000 times) in training (for internal validation) and validation cohorts (for external validation). 95% CIs for iAUC values and differences were computed by the percentile method^39^. The iAUC difference was considered statistically significant if the 95% CI of the iAUC difference did not include zero. A *p*-value of < 0.05 was considered statistically significant. Statistical analyses were performed using open source R software (version 3.3.2, https://www.r-project.org/, Supplementary materials). In addition, the radiomics quality score by Lambin et al.^11^ was evaluated to assess the overall quality of the study in a standardized form.

## Supplementary Information

Supplementary Information.

## Data Availability

The datasets generated during and/or analyzed during the current study are available from the corresponding author on reasonable request.
