# Supplementary material for: A radiomics-based model for predicting prognosis of locally advanced gastric cancer in the preoperative setting
Source: Sci Rep. 2021 Jan 21;11:1879. doi: 10.1038/s41598-021-81408-z (PMC7820605; doi:10.1038/s41598-021-81408-z)
Supplement: Supplementary file 1 — Supplementary Information. [file 41598_2021_81408_MOESM1_ESM.docx]

**A radiomics-based model for predicting prognosis of locally advanced gastric cancer in the preoperative setting**

Jaeseung Shin^1^, Joon Seok Lim^1^, Yong-Min Huh^1^, Jie-Hyun Kim^2^, Woo Jin Hyung^3^, Jae-Joon Chung^4^, Kyunghwa Han^1^, Sungwon Kim^1^

^1^Department of Radiology and Research Institute of Radiological Science, Severance Hospital, Yonsei University College of Medicine

^2^Department of Internal Medicine, Gangnam Severance Hospital, Yonsei University College of Medicine

^3^Department of Surgery, Yonsei University College of Medicine, Seoul, South Korea

^4^Department of Radiology, Gangnam Severance Hospital, Yonsei University College of Medicine, Seoul, Korea.

Correspondence and requests for materials should be addressed to S.K. (dinbe@yuhs.ac).

# SUPPLEMENTARY MATERIALS

## Radiomics Feature extraction process

PyRadiomics version 2.0.0 [https://pyradiomics.readthedocs.io/en/2.0.0/] was used for the analysis. Gray value discretization was performed to a fixed bin width of 10 bins. No normalization was performed in PyRadiomics. For the wavelet-filter, stationary wavelet transform was applied using the “coif1” (coiflet-1) wavelet function. Each image was filtered using either a high band-pass filter or low bandpass filter in x and y directions, yielding 4 different combinations of decompositions. First order features, Gray level co-occurrence matrix (GLCM) features, Gray level run length matrix (GLRLM) features, Gray level size zone matrix (GLSZM) features, and Gray level dependence matrix (GLDM) features were extracted from each original (unfiltered) as well as filtered images. Combined with the nine 2D shape features extracted from the original image, a total of 438 features were yielded. The GLCM and GLRLM features were extracted for each direction separately, after which the average value over all directions was returned as the extracted feature value, with no weighting in calculation. Feature descriptions can be found in the PyRadiomics documentation (PyRadiomics feature definitions: https://pyradiomics.readthedocs.io/en/latest/features.html)

## R Software packages used for statistical analysis

The “glmnet” and “survival” package was used to perform the least absolute shrinkage and selection operator or LASSO Cox regression and survival analysis, respectively. The “rms” package was used to perform multivariate Cox regression and generate nomograms and calibration plots. The “survival ROC” package was used to perform the time-dependent receiver operating characteristic (ROC) curve analysis. The integrated area under the receiver operating characteristic curve (iAUC) values were calculated by using the “riskset ROC” package.

# SUPPLEMENTAL TABLE

**Table S1. Radiomics quality score**

| RQS criteria | | | Points |  |
| --- | --- | --- | --- | --- |
| 1 | Image protocol quality | Well-documented image protocols (for example, contrast, slice thickness, energy, etc.) and/or usage of public image protocols allow reproducibility/replicability | + 1 (if protocols are well-documented)  + 1 (if public protocol is used) | +1 |
| 2 | Multiple segmentations | Segmentation by different physicians/algorithms/software, perturbing segmentations by (random) noise, segmentation at different breathing cycles. Analyse feature robustness to segmentation variabilities | + 1 | +1 |
| 3 | Phantom study on all scanners | Detect inter-scanner differences and vendor-dependent features. Analyse feature robustness to these sources of variability | + 1 | 0 |
| 4 | Imaging at multiple time points | Collect images of individuals at additional time points. Analyse feature robustness to temporal variabilities (for example, organ movement, organ expansion/ shrinkage) | + 1 | 0 |
| 5 | Feature reduction or adjustment for multiple testing | Decreases the risk of overfitting. Overfitting is inevitable if the number of features exceeds the number of samples. Consider feature robustness when selecting features | - 3 (if neither measure is implemented) + 3 (if either measure is implemented) | +3 |
| 6 | Multivariable analysis with non-radiomics features | (for example, EGFR mutation) - is expected to provide a more holistic model. Permits correlating/inferencing between radiomics and non radiomics features | +1 | +1 |
| 7 | Detect and discuss biological correlates | Demonstration of phenotypic differences (possibly associated with underlying gene–protein expression patterns) deepens understanding of radiomics and biology | +1 | 0 |
| 8 | Cut-off analyses | Determine risk groups by either the median, a previously published cut-off or report a continuous risk variable. Reduces the risk of reporting overly optimistic results | +1 | +1 |
| 9 | Discrimination statistics | Report discrimination statistics (for example, C‑statistic, ROC curve, AUC) and their statistical significance (for example, p‑values, confidence intervals). One can also apply resampling method (for example, bootstrapping, cross-validation) | + 1 (if a discrimination statistic and its statistical significance are reported) + 1 (if a resampling method technique is also applied) | +1 |
| 10 | Calibration statistics | Report calibration statistics (for example, Calibration-in‑the-large/slope, calibration plots) and their statistical significance (for example, *P*‑values, confidence intervals). One can also apply resampling method (for example, bootstrapping, cross-validation) | + 1 (if a calibration statistic and its statistical significance are reported) + 1 (if a resampling method technique is also applied) | +1 |
| 11 | Prospective study registered in a trial database | Provides the highest level of evidence supporting the clinical validity and usefulness of the radiomics biomarker | + 7 (for prospective validation of a radiomics signature in an appropriate trial) | 0 |
| 12 | Validation | The validation is performed without retraining and without adaptation of the cut-off value, provides crucial information with regards to credible clinical performance | - 5 (if validation is missing)  + 2 (if validation is based on a dataset from the same institute)  + 3 (if validation is based on a dataset from another institute)  + 4 (if validation is based on two datasets from two distinct institutes)  + 4 (if the study validates a previously published signature)  + 5 (if validation is based on three or more datasets from distinct institutes) | +3 |
| 13 | Comparison to ‘gold standard’ | Assess the extent to which the model agrees with/is superior to the current ‘gold standard’ method (for example, TNM-staging for survival prediction). This comparison shows the added value of radiomics | +2 | +2 |
| 14 | Potential clinical utility | Report on the current and potential application of the model in a clinical setting (for example, decision curve analysis). | +2 | 0 |
| 15 | Cost-effectiveness analysis | Report on the cost-effectiveness of the clinical application (for example, QALYs generated) | +1 | 0 |
| 16 | Open science and data | Make code and data publicly available. Open science facilitates knowledge transfer and reproducibility of the study | + 1 (if scans are open source) + 1 (if region of interest segmentations are open source) + 1 (if code is open source) + 1 (if radiomics features are calculated on a set of representative ROIs and the calculated features and representative ROIs are open source) | +1 |
|  |  |  | Total points (36 = 100%) | 15 (41.7%) |

Table S2. Details of CT acquisition Parameters

| Parameters | Sensation 16 | Sensation 64 | Lightspeed VCT |
| --- | --- | --- | --- |
| No. of channels | 16 | 64 | 64 |
| Section collimation^*^ | 16 x 0.75 | 64 x 0.6 | 64 x 0.625 |
| Slice thickness (mm) | 3 | 3 | 3 |
| Pitch | 1 | 0.6 | 0.9840 |
| Tube current (mAs)^†^ | 160 | 160 | 100–300^‡^ |
| Rotation time (sec) | 0.5 | 0.5 | 0.5 |
| Table speed (mm per rotation) | 24.0 | 24.0 | 24.0 |
| Tube voltage (kV) | 120 | 120 | 120 |
| Kernel | B30f/B31f | | Standard |
| Matrix | 512 x 512 | 512 x 512 | 512 x 512 |
| Following CT scanners were used: Sensation 16 and Sensation 64 (Siemens Healthineers); and Lightspeed VCT (GE Healthcare). ^*^Number of detector rows times section thickness (mm), ^†^Reference milliampere-seconds, ^‡^AutomA was set between 100 and 300 mA with a noise index of 15. CT = computed tomography | | | |

**Table S3.** List of radiomic features extracted from the computed tomography images and Inter-slice and Inter-reader intraclass coefficient correlation.

| Feature family | Feature class | Inter-slice ICC | | | | | Inter-reader ICC | | | | |
| --- | --- | --- | --- | --- | --- | --- | --- | --- | --- | --- | --- |
|  |  | Original | Wavelet-filtered | | | | Original | Wavelet-filtered | | | |
|  |  |  | LH | HL | HH | LL |  | LH | HL | HH | LL |
| Shape Features (2D) (8 features) | Elongation | 0.924 | - | - | - | - | 0.809 | - | - | - | - |
|  | Major axis | 0.928 | - | - | - | - | 0.848 | - | - | - | - |
|  | Minor axis | 0.984 | - | - | - | - | 0.922 | - | - | - | - |
|  | Maximum 2D diameter (column) | 0.934 | - | - | - | - | 0.900 | - | - | - | - |
|  | Maximum 2D diameter (row) | 0.982 | - | - | - | - | 0.832 | - | - | - | - |
|  | Maximum 2D diameter (slice) | 0.920 | - | - | - | - | 0.835 | - | - | - | - |
|  | Sphericity | 0.975 | - | - | - | - | 0.875 | - | - | - | - |
|  | Surface area | 0.967 | - | - | - | - | 0.909 | - | - | - | - |
| First order features (18 features) | Energy | 0.971 | 0.968 | 0.968 | 0.967 | 0.974 | 0.911 | 0.910 | 0.909 | 0.909 | 0.912 |
|  | Total energy | 0.971 | 0.968 | 0.968 | 0.967 | 0.974 | 0.911 | 0.910 | 0.909 | 0.909 | 0.912 |
|  | Entropy^*^ | 0.691 | 0.949 | 0.979 | 0.995 | 0.727 | 0.810 | 0.828 | 0.863 | 0.918 | 0.793 |
|  | Minimum | -0.065 | 0.924 | 0.961 | 0.843 | -0.104 | 0.004 | 0.747 | 0.731 | 0.801 | 0.010 |
|  | Maximum | 0.980 | 0.362 | 0.668 | 0.898 | 0.971 | 0.843 | 0.482 | 0.418 | 0.746 | 0.827 |
|  | 10th percentile | 0.358 | 0.963 | 0.978 | 0.994 | 0.387 | 0.712 | 0.773 | 0.814 | 0.965 | 0.731 |
|  | 90th percentile | 0.993 | 0.891 | 0.994 | 0.992 | 0.995 | 0.933 | 0.930 | 0.891 | 0.926 | 0.922 |
|  | Mean | 0.845 | 0.944 | 0.980 | 0.804 | 0.842 | 0.891 | 0.817 | 0.814 | 0.630 | 0.892 |
|  | Median | 0.914 | 0.914 | 0.965 | 0.722 | 0.919 | 0.907 | 0.638 | 0.645 | 0.393 | 0.905 |
|  | Interquartile range | 0.776 | 0.921 | 0.977 | 0.998 | 0.776 | 0.756 | 0.752 | 0.831 | 0.963 | 0.751 |
|  | Range | 0.050 | 0.821 | 0.930 | 0.910 | 0.009 | 0.118 | 0.709 | 0.622 | 0.824 | 0.131 |
|  | Mean absolute deviation | 0.536 | 0.962 | 0.982 | 0.998 | 0.552 | 0.750 | 0.800 | 0.807 | 0.941 | 0.759 |
|  | Robust mean absolute deviation | 0.764 | 0.930 | 0.982 | 0.998 | 0.769 | 0.772 | 0.764 | 0.836 | 0.961 | 0.778 |
|  | Root mean squared | 0.867 | 0.942 | 0.980 | 0.802 | 0.882 | 0.897 | 0.816 | 0.815 | 0.616 | 0.902 |
|  | Skewness | 0.174 | 0.891 | 0.879 | 0.849 | 0.199 | 0.345 | 0.734 | 0.765 | 0.646 | 0.367 |
|  | Kurtosis^†^ | 0.006 | 0.953 | 0.941 | 0.913 | 0.007 | 0.020 | 0.838 | 0.699 | 0.741 | 0.022 |
|  | Variance | 0.792 | 0.960 | 0.983 | 0.995 | 0.821 | 0.793 | 0.831 | 0.877 | 0.920 | 0.789 |
|  | Uniformity^‡^ | 0.134 | 0.968 | 0.978 | 0.998 | 0.143 | 0.414 | 0.790 | 0.715 | 0.927 | 0.433 |
| Gray level co-occurrence matrix features (GLCM) (22 features) | Autocorrelation | 0.005 | 0.942 | 0.966 | 0.855 | -0.001 | 0.034 | 0.697 | 0.664 | 0.721 | 0.035 |
|  | Joint average | 0.055 | 0.913 | 0.955 | 0.820 | 0.018 | 0.124 | 0.730 | 0.731 | 0.741 | 0.123 |
|  | Cluster Prominence | 0.001 | 0.983 | 0.974 | 0.998 | 0.001 | -0.002 | 0.700 | 0.534 | 0.749 | -0.001 |
|  | Cluster Shade | 0.008 | 0.978 | 0.975 | 0.886 | 0.008 | 0.016 | 0.758 | 0.635 | 0.360 | 0.019 |
|  | Cluster Tendency | 0.139 | 0.972 | 0.977 | 0.998 | 0.144 | 0.455 | 0.803 | 0.729 | 0.906 | 0.453 |
|  | Contrast | 0.412 | 0.967 | 0.984 | 0.998 | 0.383 | 0.700 | 0.847 | 0.751 | 0.950 | 0.695 |
|  | Correlation | 0.753 | 0.955 | 0.981 | 0.980 | 0.736 | 0.580 | 0.832 | 0.867 | 0.855 | 0.569 |
|  | Difference average | 0.810 | 0.980 | 0.989 | 0.998 | 0.790 | 0.890 | 0.892 | 0.864 | 0.959 | 0.866 |
|  | Difference entropy | 0.844 | 0.972 | 0.980 | 0.997 | 0.865 | 0.905 | 0.881 | 0.855 | 0.940 | 0.891 |
|  | Difference variance | 0.211 | 0.936 | 0.967 | 0.998 | 0.249 | 0.450 | 0.759 | 0.635 | 0.915 | 0.548 |
|  | Joint energy^§^ | 0.864 | 0.970 | 0.983 | 0.995 | 0.877 | 0.786 | 0.829 | 0.883 | 0.926 | 0.747 |
|  | Joint entropy | 0.785 | 0.956 | 0.981 | 0.996 | 0.772 | 0.839 | 0.864 | 0.884 | 0.937 | 0.750 |
|  | Informational measure of correlation 1 | 0.820 | 0.964 | 0.968 | 0.956 | 0.857 | 0.775 | 0.795 | 0.820 | 0.513 | 0.766 |
|  | Informational measure of correlation 2 | 0.858 | 0.934 | 0.972 | 0.972 | 0.839 | 0.836 | 0.800 | 0.831 | 0.609 | 0.838 |
|  | Inverse difference moment | 0.954 | 0.988 | 0.991 | 0.998 | 0.961 | 0.895 | 0.900 | 0.901 | 0.953 | 0.869 |
|  | Inverse difference moment normalized | 0.706 | 0.878 | 0.937 | 0.738 | 0.807 | 0.645 | 0.766 | 0.737 | 0.447 | 0.691 |
|  | Inverse difference | 0.949 | 0.987 | 0.991 | 0.998 | 0.953 | 0.895 | 0.899 | 0.901 | 0.952 | 0.874 |
|  | Inverse difference normalized | 0.564 | 0.887 | 0.945 | 0.784 | 0.683 | 0.618 | 0.793 | 0.746 | 0.645 | 0.681 |
|  | Inverse variance | 0.931 | 0.980 | 0.985 | 0.996 | 0.956 | 0.912 | 0.883 | 0.814 | 0.944 | 0.872 |
|  | Maximum probability^‖^ | 0.861 | 0.978 | 0.992 | 0.996 | 0.885 | 0.666 | 0.815 | 0.880 | 0.936 | 0.623 |
|  | Sum entropy | 0.706 | 0.931 | 0.972 | 0.994 | 0.709 | 0.758 | 0.797 | 0.871 | 0.910 | 0.734 |
|  | Sum of squares^¶^ | 0.155 | 0.971 | 0.979 | 0.998 | 0.159 | 0.484 | 0.796 | 0.727 | 0.933 | 0.487 |
| Gray level run length matrix features (GLRLM) (16 features) | Short run emphasis | 0.967 | 0.985 | 0.989 | 0.997 | 0.968 | 0.839 | 0.870 | 0.900 | 0.941 | 0.843 |
|  | Long run emphasis | 0.978 | 0.990 | 0.989 | 0.995 | 0.974 | 0.849 | 0.865 | 0.917 | 0.922 | 0.842 |
|  | Gray level non-uniformity | 0.983 | 0.972 | 0.968 | 0.962 | 0.985 | 0.943 | 0.923 | 0.911 | 0.903 | 0.944 |
|  | Gray level non-uniformity normalized | 0.764 | 0.956 | 0.977 | 0.994 | 0.804 | 0.807 | 0.829 | 0.865 | 0.904 | 0.798 |
|  | Run length non-uniformity | 0.966 | 0.975 | 0.977 | 0.988 | 0.960 | 0.904 | 0.915 | 0.914 | 0.936 | 0.900 |
|  | Run length non-uniformity normalized | 0.967 | 0.986 | 0.991 | 0.997 | 0.969 | 0.840 | 0.871 | 0.895 | 0.944 | 0.840 |
|  | Run percentage | 0.975 | 0.989 | 0.991 | 0.997 | 0.973 | 0.853 | 0.879 | 0.903 | 0.938 | 0.844 |
|  | Gray level variance | 0.101 | 0.968 | 0.976 | 0.998 | 0.126 | 0.323 | 0.789 | 0.707 | 0.914 | 0.385 |
|  | Run variance | 0.983 | 0.990 | 0.989 | 0.992 | 0.977 | 0.851 | 0.841 | 0.915 | 0.894 | 0.828 |
|  | Run entropy | 0.596 | 0.926 | 0.959 | 0.972 | 0.590 | 0.710 | 0.774 | 0.758 | 0.760 | 0.687 |
|  | Low gray level run emphasis | -0.053 | 0.850 | 0.938 | 0.525 | 0.097 | 0.382 | 0.437 | 0.631 | 0.444 | 0.421 |
|  | High gray level run emphasis | 0.000 | 0.940 | 0.965 | 0.868 | -0.006 | 0.027 | 0.697 | 0.662 | 0.733 | 0.029 |
|  | Short run low gray level emphasis | -0.009 | 0.840 | 0.928 | 0.557 | 0.123 | 0.396 | 0.412 | 0.632 | 0.415 | 0.439 |
|  | Short run high gray level emphasis | 0.003 | 0.937 | 0.964 | 0.913 | -0.001 | 0.032 | 0.690 | 0.654 | 0.779 | 0.035 |
|  | Long run low gray level emphasis | -0.178 | 0.893 | 0.958 | 0.516 | -0.011 | 0.338 | 0.554 | 0.694 | 0.571 | 0.337 |
|  | Long run high gray level emphasis | 0.000 | 0.953 | 0.967 | 0.599 | -0.017 | 0.028 | 0.747 | 0.692 | 0.555 | 0.018 |
| Gray level size zone matrix features (GLSZM) (16 features) | Small area emphasis | 0.850 | 0.961 | 0.907 | 0.895 | 0.930 | 0.802 | 0.733 | 0.719 | 0.672 | 0.828 |
|  | Large area emphasis | 0.973 | 0.943 | 0.992 | 0.940 | 0.966 | 0.740 | 0.698 | 0.712 | 0.770 | 0.803 |
|  | Gray level non-uniformity | 0.980 | 0.979 | 0.980 | 0.992 | 0.982 | 0.939 | 0.926 | 0.914 | 0.942 | 0.942 |
|  | Gray level non-uniformity normalized | 0.624 | 0.944 | 0.965 | 0.874 | 0.728 | 0.789 | 0.756 | 0.822 | 0.686 | 0.788 |
|  | Size-zone non-uniformity | 0.954 | 0.989 | 0.983 | 0.996 | 0.931 | 0.896 | 0.928 | 0.929 | 0.950 | 0.861 |
|  | Size-zone non-uniformity normalized | 0.868 | 0.962 | 0.924 | 0.906 | 0.935 | 0.802 | 0.725 | 0.732 | 0.687 | 0.822 |
|  | Zone percentage | 0.962 | 0.987 | 0.990 | 0.996 | 0.967 | 0.895 | 0.868 | 0.878 | 0.952 | 0.859 |
|  | Gray level variance | 0.044 | 0.949 | 0.967 | 0.969 | 0.086 | 0.137 | 0.740 | 0.681 | 0.810 | 0.263 |
|  | Zone variance | 0.975 | 0.940 | 0.992 | 0.940 | 0.965 | 0.705 | 0.686 | 0.704 | 0.769 | 0.758 |
|  | Zone entropy | 0.753 | 0.904 | 0.928 | 0.903 | 0.730 | 0.732 | 0.678 | 0.661 | 0.763 | 0.732 |
|  | Low gray level zone emphasis | -0.026 | 0.842 | 0.937 | 0.593 | 0.100 | 0.423 | 0.462 | 0.661 | 0.439 | 0.438 |
|  | High gray level zone emphasis | -0.005 | 0.938 | 0.961 | 0.864 | -0.009 | 0.019 | 0.694 | 0.659 | 0.714 | 0.024 |
|  | Small area low gray level zone emphasis | 0.130 | 0.797 | 0.888 | 0.576 | 0.172 | 0.456 | 0.412 | 0.651 | 0.445 | 0.474 |
|  | Small area high gray level zone emphasis | 0.009 | 0.931 | 0.954 | 0.868 | 0.009 | 0.035 | 0.686 | 0.642 | 0.707 | 0.047 |
|  | Large area low gray level zone emphasis | -0.011 | 0.917 | 0.859 | 0.493 | -0.063 | 0.510 | 0.404 | 0.461 | 0.635 | 0.289 |
|  | Large area high gray level zone emphasis | 0.007 | 0.971 | 0.988 | 0.940 | -0.011 | 0.108 | 0.681 | 0.865 | 0.814 | 0.060 |
| Gray level dependence matrix features (GLDM) (14 features) | Small dependence emphasis | 0.950 | 0.989 | 0.987 | 0.997 | 0.959 | 0.890 | 0.857 | 0.871 | 0.954 | 0.854 |
|  | Large dependence emphasis | 0.980 | 0.989 | 0.990 | 0.996 | 0.977 | 0.821 | 0.873 | 0.909 | 0.932 | 0.821 |
|  | Gray level non-uniformity | 0.983 | 0.972 | 0.964 | 0.960 | 0.986 | 0.942 | 0.916 | 0.910 | 0.903 | 0.943 |
|  | Dependence non-uniformity | 0.968 | 0.979 | 0.973 | 0.970 | 0.956 | 0.907 | 0.919 | 0.915 | 0.917 | 0.895 |
|  | Dependence non-uniformity normalized | 0.985 | 0.993 | 0.990 | 0.990 | 0.976 | 0.847 | 0.875 | 0.875 | 0.870 | 0.816 |
|  | Gray level variance | 0.135 | 0.968 | 0.978 | 0.998 | 0.143 | 0.415 | 0.792 | 0.715 | 0.928 | 0.434 |
|  | Dependence variance | 0.986 | 0.989 | 0.980 | 0.982 | 0.986 | 0.702 | 0.830 | 0.851 | 0.806 | 0.706 |
|  | Dependence entropy | 0.702 | 0.884 | 0.959 | 0.992 | 0.704 | 0.674 | 0.669 | 0.791 | 0.894 | 0.669 |
|  | Low gray level emphasis | -0.055 | 0.851 | 0.939 | 0.513 | 0.092 | 0.365 | 0.429 | 0.615 | 0.463 | 0.407 |
|  | High gray level emphasis | 0.002 | 0.941 | 0.966 | 0.868 | -0.004 | 0.030 | 0.698 | 0.663 | 0.735 | 0.031 |
|  | Small dependence low gray level emphasis | 0.275 | 0.802 | 0.806 | 0.753 | 0.257 | 0.492 | 0.365 | 0.647 | 0.462 | 0.512 |
|  | Small dependence high gray level emphasis | 0.046 | 0.938 | 0.959 | 0.979 | 0.037 | 0.083 | 0.695 | 0.616 | 0.880 | 0.091 |
|  | Large dependence low gray level emphasis | -0.231 | 0.902 | 0.961 | 0.534 | -0.122 | 0.307 | 0.589 | 0.675 | 0.562 | 0.148 |
|  | Large dependence high gray level emphasis | 0.008 | 0.959 | 0.971 | 0.584 | -0.017 | 0.036 | 0.759 | 0.705 | 0.523 | 0.020 |

ICC, Intraclass correlation coefficient; LL, Low-Low pass filter; LH, Low-High pass filter; HH, High-High pass filter; LL, Low-Low pass filter
* Defined by image biomarker standardization initiative (IBSI) as Intensity Histogram Entropy
† The IBSI feature definition implements excess kurtosis, where kurtosis is corrected by -3, yielding 0 for normal distributions. The PyRadiomics kurtosis is not corrected, yielding a value 3 higher than the IBSI kurtosis.
‡ Defined by IBSI as Intensity Histogram Uniformity
§ Defined by IBSI as Angular Second Moment.
‖ Defined by IBSI as Joint maximum
¶ Defined by IBSI as Joint Variance

**Table S4. Checklist for reporting on radiomics studies by image biomarker standardization initiative guideline**

| Topic | Modality | Item | Description | Page |
| --- | --- | --- | --- | --- |
| Patient | | | | |
| Region of interest |  | 1 | Describe the region of interest that is being imaged. | 18 |
| Patient preparation |  | 2a | Describe specific instructions given to patients prior to image acquisition, e.g. fasting prior to imaging. | 17 |
|  |  | 2b | Describe administration of drugs to the patient prior to image acquisition, e.g. muscle relaxants. | 17 |
|  |  | 2c | Describe the use of specific equipment for patient comfort during scanning, e.g. ear plugs. | N/A |
| Radioactive tracer | PET, SPECT | 3a | Describe which radioactive tracer was administered to the patient, e.g. 18F-FDG. | N/A |
|  | PET, SPECT | 3b | Describe the administration method. | N/A |
|  | PET, SPECT | 3c | Describe the injected activity of the radioactive tracer at administration. | N/A |
|  | PET, SPECT | 3d | Describe the uptake time prior to image acquisition. | N/A |
|  | PET, SPECT | 3e | Describe how competing substance levels were controlled. | N/A |
| Contrast agent |  | 4a | Describe which contrast agent was administered to the patient. | 17 |
|  |  | 4b | Describe the administration method. | 17 |
|  |  | 4c | Describe the injected quantity of contrast agent. | 17 |
|  |  | 4d | Describe the uptake time prior to image acquisition. | 17 |
|  |  | 4e | Describe how competing substance levels were controlled. | N/A |
| Comorbidities |  | 5 | Describe if the patients have comorbidities that affect imaging. | N/A |
| Acquisition | | | | |
| Acquisition protocol |  | 6 | Describe whether a standard imaging protocol was used, and where its description may be found. | 17, Sup |
| Scanner type |  | 7 | Describe the scanner type(s) and vendor(s) used in the study. | 17, Sup |
| Imaging modality |  | 8 | Clearly state the imaging modality that was used in the study, e.g. CT, MRI. | 17, Sup |
| Static/dynamic scans |  | 9a | State if the scans were static or dynamic. | 17 |
|  | Dynamic scans | 9b | Describe the acquisition time per time frame. | N/A |
|  | Dynamic scans | 9c | Describe any temporal modelling technique that was used. | N/A |
| Scanner calibration |  | 10 | Describe how and when the scanner was calibrated. | N/A |
| Patient instructions |  | 11 | Describe specific instructions given to the patient during acquisition, e.g. breath holding. | N/A |
| Anatomical motion correction |  | 12 | Describe the method used to minimise the effect of anatomical motion. | N/A |
| Scan duration |  | 13 | Describe the duration of the complete scan or the time per bed position. | 17 |
| Tube voltage | CT | 14 | Describe the peak kilo voltage output of the X-ray source. | 17, Sup |
| Tube current | CT | 15 | Describe the tube current in mA. | 17, Sup |
| Time-of-flight | PET | 16 | State if scanner time-of-flight capabilities are used during acquisition. | N/A |
| RF coil | MRI | 17 | Describe what kind RF coil used for acquisition, incl. vendor. | N/A |
| Scanning sequence | MRI | 18a | Describe which scanning sequence was acquired. | N/A |
|  | MRI | 18b | Describe which sequence variant was acquired. | N/A |
|  | MRI | 18c | Describe which scan options apply to the current sequence, e.g. flow compensation, cardiac gating. | N/A |
| Repetition time | MRI | 19 | Describe the time in ms between subsequent pulse sequences. | N/A |
| Echo time | MRI | 20 | Describe the echo time in ms. | N/A |
| Echo train length | MRI | 21 | Describe the number of lines in k-space that are acquired per excitation pulse. | N/A |
| Inversion time | MRI | 22 | Describe the time in ms between the middle of the inverting RF pulse to the middle of the excitation pulse. | N/A |
| Flip angle | MRI | 23 | Describe the flip angle produced by the RF pulses. | N/A |
| Acquisition type | MRI | 24 | Describe the acquisition type of the MRI scan, e.g. 3D. | N/A |
| k-space traversal | MRI | 25 | Describe the acquisition trajectory of the k-space. | N/A |
| Number of averages/ excitations | MRI | 26 | Describe the number of times each point in k-space is sampled. | N/A |
| Magnetic field strength | MRI | 27 | Describe the nominal strength of the MR magnetic field. | N/A |
| Reconstruction | | | | |
| In-plane resolution |  | 28 | Describe the distance between pixels, or alternatively the field of view and matrix size. | 17, Sup |
| Image slice thickness |  | 29 | Describe the slice thickness. | 17, Sup |
| Image slice spacing |  | 30 | Describe the distance between image slices. | 17, Sup |
| Convolution kernel | CT | 31a | Describe the convolution kernel used to reconstruct the image. | 17, Sup |
|  | CT | 31b | Describe settings pertaining to iterative reconstruction algorithms. | 17, Sup |
| Exposure | CT | 31c | Describe the exposure (in mAs) in slices containing the region of interest. | 17, Sup |
| Reconstruction method | PET | 32a | Describe which reconstruction method was used, e.g. 3D OSEM. | N/A |
|  | PET | 32b | Describe the number of iterations for iterative reconstruction. | N/A |
|  | PET | 32c | Describe the number of subsets for iterative reconstruction. | N/A |
| Point spread function modelling | PET | 33 | Describe if and how point-spread function modelling was performed. | N/A |
| Image corrections | PET | 34a | Describe if and how attenuation correction was performed. | N/A |
|  | PET | 34b | Describe if and how other forms of correction were performed, e.g. scatter correction, randoms correction, dead time correction etc. | N/A |
| Reconstruction method | MRI | 35a | Describe the reconstruction method used to reconstruct the image from the k-space information. | N/A |
|  | MRI | 35b | Describe any artifact suppression methods used during reconstruction to suppress artifacts due to undersampling of k-space. | N/A |
| Diffusion-weigh ted imaging | DWI-MRI | 36 | Describe the b-values used for diffusion-weigh ting. | N/A |
| Image registration | | | | |
| Registration method |  | 37 | Describe the method used to register multi-modality imaging. | N/A |
| Image processing-data conversion |  |  |  |  |
| SUV normalisation | PET | 38 | Describe which standardised uptake value (SUV) normalisation method is used. | N/A |
| ADC computation | DWI-MRI | 39 | Describe how apparent diffusion coefficient (ADC) values were calculated. | N/A |
| Other data conversions |  | 40 | Describe any other conversions that are performed to generate e.g. perfusion maps. | N/A |
| Image processing-postacquisition processing | | | | |
| Anti-aliasing |  | 41 | Describe the method used to deal with anti-aliasing when down-sampling during interpolation. | N/A |
| Noise suppression |  | 42 | Describe methods used to suppress image noise. | N/A |
| Post-reconstruc tion smoothing filter | PET | 43 | Describe the width of the Gaussian filter (FWHM) to spatially smooth intensities. | N/A |
| Skull stripping | MRI (brain) | 44 | Describe method used to perform skull stripping. | N/A |
| Non-uniformity correction | MRI | 45 | Describe the method and settings used to perform non-uniformity correction. | N/A |
| Intensity normalisation |  | 46 | Describe the method and settings used to normalise intensity distributions within a patient or patient cohort. | Sup |
| Other post-acquisitio n processing methods |  | 47 | Describe any other methods that were used to process the image and are not mentioned separately in this list. | 18, Sup |
| Segmentation | | | | |
| Segmentation method |  | 48a | Describe how regions of interest were segmented, e.g. manually. | 18-19, Sup |
|  |  | 48b | Describe the number of experts, their expertise and consensus strategies for manual delineation. | 19 |
|  |  | 48c | Describe methods and settings used for semi-automatic and fully automatic segmentation. | 18-19 |
|  |  | 48d | Describe which image was used to define segmentation in case of multi-modality imaging. | N/A |
| Conversion to mask |  | 49 | Describe the method used to convert polygonal or mesh-based segmentations to a voxel-based mask. | N/A |
| Image processing-image interpolation | | | | |
| Interpolation method |  | 50a | Describe which interpolation algorithm was used to interpolate the image. | 18 |
|  |  | 50b | Describe how the position of the interpolation grid was defined, e.g. align by center. | 18 |
|  |  | 50c | Describe how the dimensions of the interpolation grid were defined, e.g. rounded to nearest integer. | 18 |
|  |  | 50d | Describe how extrapolation beyond the original image was handled. | N/A |
| Voxel dimensions |  | 51 | Describe the size of the interpolated voxels. | 18 |
| Intensity rounding | CT | 52 | Describe how fractional Hounsfield Units are rounded to integer values after interpolation. | N/A |
| Image processing-ROI interpolation | | | | |
| Interpolation method |  | 53 | Describe which interpolation algorithm was used to interpolate the region of interest mask. | 18 |
| Partially masked voxels |  | 54 | Describe how partially masked voxels after interpolation are handled. | N/A |
| Image processing-resegmentation |  |  |  |  |
| Re-segmentation methods |  | 55 | Describe which methods and settings are used to re-segment the ROI intensity mask. | N/A |
| Image processing-discretization | | | | |
| Discretisation method |  | 56a | Describe the method used to discretise image intensities. | Sup |
|  |  | 56b | Describe the number of bins (FBN) or the bin size (FBS) used for discretisation. | Sup |
|  |  | 56c | Describe the lowest intensity in the first bin for FBS discretisation. | Sup |
| Image processing-image transformation | | | | |
| Image filter |  | 57 | Describe the methods and settings used to filter images, e.g. Laplacian-of-Gaussian. | 19, Sup |
| Radiomics feature computation | | | | |
| Biomarker set |  | 58 | Describe which set of image biomarkers is computed and refer to their definitions or provide these. | Sup |
| IBSI compliance |  | 59 | State if the software used to extract the set of image biomarkers is compliant with the IBSI benchmarks. | Sup |
| Robustness |  | 60 | Describe how robustness of the image biomarkers was assessed, e.g. test-retest analysis. | 19 |
| Software availability |  | 61 | Describe which software and version was used to compute image biomarkers. | 18-19 |
| Radiomics feature computation-texture parameters | | | | |
| Texture matrix aggregation |  | 62 | Define how texture-matrix based biomarkers were computed from underlying texture matrices. | Sup |
| Distance weighting |  | 63 | Define how CM, RLM, NGTDM and NGLDM weight distances, e.g. no weighting. | Sup |
| CM symmetry |  | 64 | Define whether symmetric or asymmetric co-occurrence matrices were computed. | Sup |
| CM distance |  | 65 | Define the (Chebyshev) distance at which co-occurrence of intensities is determined, e.g. 1. | Sup |
| SZM linkage distance |  | 66 | Define the distance and distance norm for which voxels with the same intensity are considered to belong to the same zone for the purpose of constructing an SZM, e.g. Chebyshev distance of 1. | Sup |
| DZM linkage distance |  | 67 | Define the distance and distance norm for which voxels with the same intensity are considered to belong to the same zone for the purpose of constructing a DZM, e.g. Chebyshev distance of 1. | Sup |
| DZM zone distance norm |  | 68 | Define the distance norm for determining the distance of zones to the border of the ROI, e.g. Manhattan distance. | Sup |
| NGTDM distance |  | 69 | Define the neighbourhood distance and distance norm for the NGTDM, e.g. Chebyshev distance of 1. | N/A |
| NGLDM distance |  | 70 | Define the neighbourhood distance and distance norm for the NGLDM, e.g. Chebyshev distance of 1. | N/A |
| NGLDM coarseness |  | 71 | Define the coarseness parameter for the NGLDM, e.g. 0. | N/A |
| Machine learning and radiomics analysis | | | | |
| Diagnostic and prognostic modelling |  | 72 | See the TRIPOD guidelines for reporting on diagnostic and prognostic modelling. | 20-21 |
| Comparison with known factors |  | 73 | Describe where performance of radiomics models is compared with known (clinical) factors. | 20 |
| Multicollinearity |  | 74 | Describe where the multicollineari ty between image biomarkers in the signature is assessed. | 14, Sup |
| Model availability |  | 75 | Describe where radiomics models with the necessary pre-processing information may be found. | 22 |
| Data availability |  | 76 | Describe where imaging data and relevant meta-data used in the study may be found. | 22 |

N/A, Not applicable; Sup, Supplementary material

# SUPPLEMENTAL FIGURES


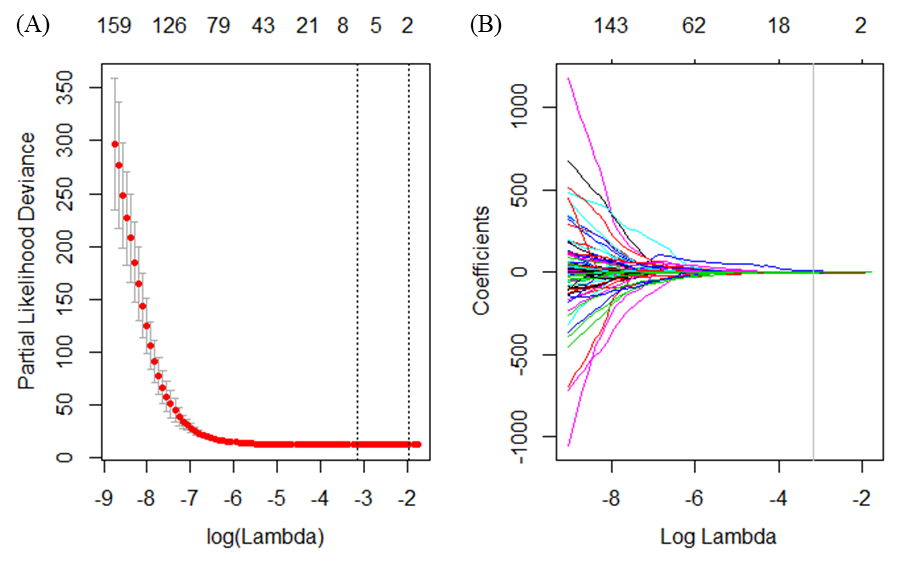


**Figure S1. Texture feature selection using the least absolute shrinkage and selection operator (LASSO) Cox regression model.** (A) Tuning parameter (λ) selection in the LASSO model used 10-fold cross-validation via minimum criteria. (B) LASSO coefficient profiles of the 438 texture features. R software (version 3.3.2, <https://www.r-project.org>) was used to draw.


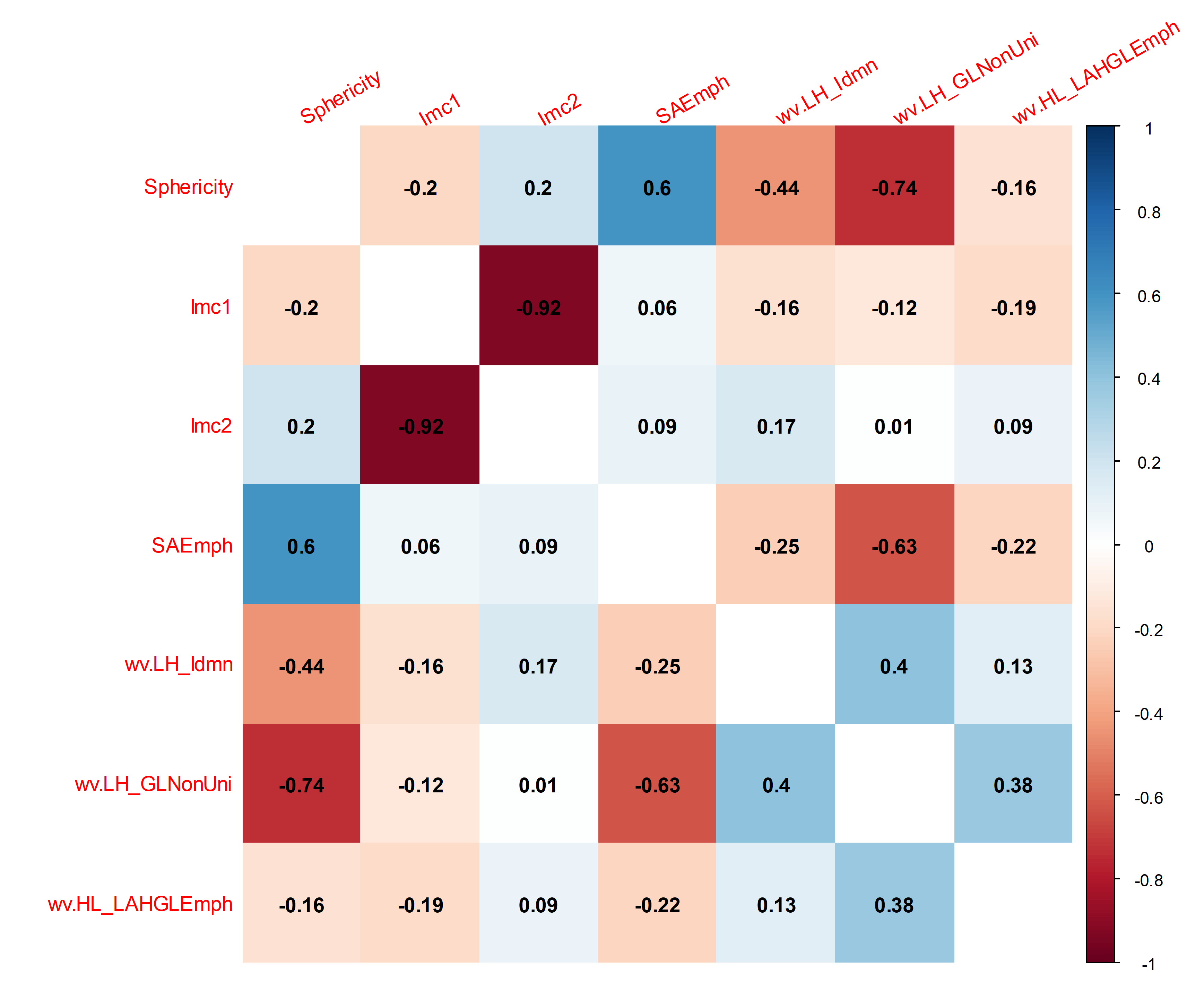


**Figure S2. Correlation matrix of selected radiomics features.** R software (version 3.3.2, <https://www.r-project.org>) was used to draw.
GLNonUni, GrayLevelNonUniformity; HL, Hihg-low pass filter; Idmn, Inverse difference moment normalized; IMC, informational measure of correlation; LAHGLEmph, LargeAreaHighGrayLevelEmphasis; LH, Low-high pass filter; SAEmph, SmallAreaEmphasis; wv, wavelet;


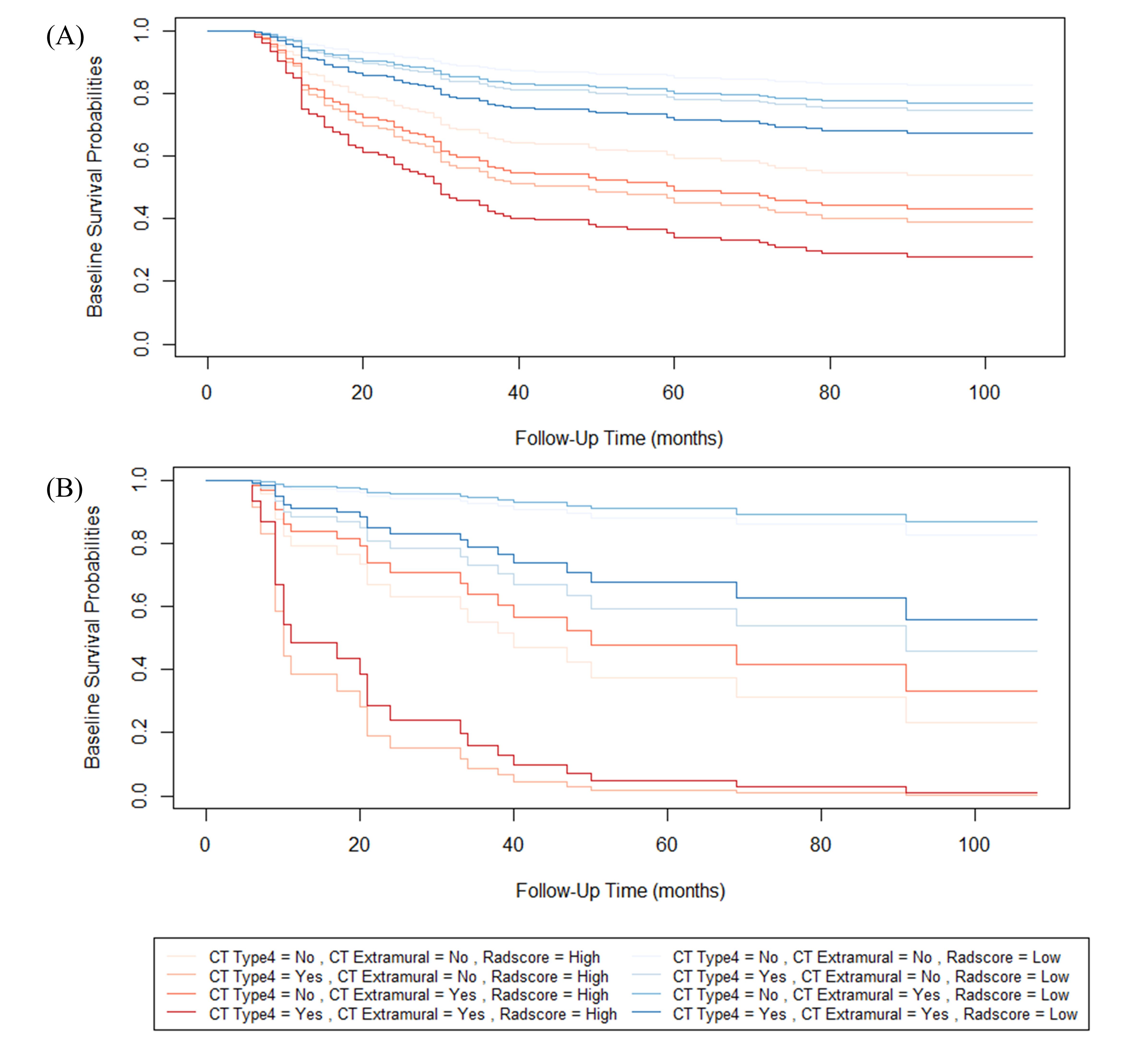


**Figure S3. Estimates of the baseline survival function in (A) training cohort and (B) validation cohort.** R software (version 3.3.2, <https://www.r-project.org>) was used to draw.

**
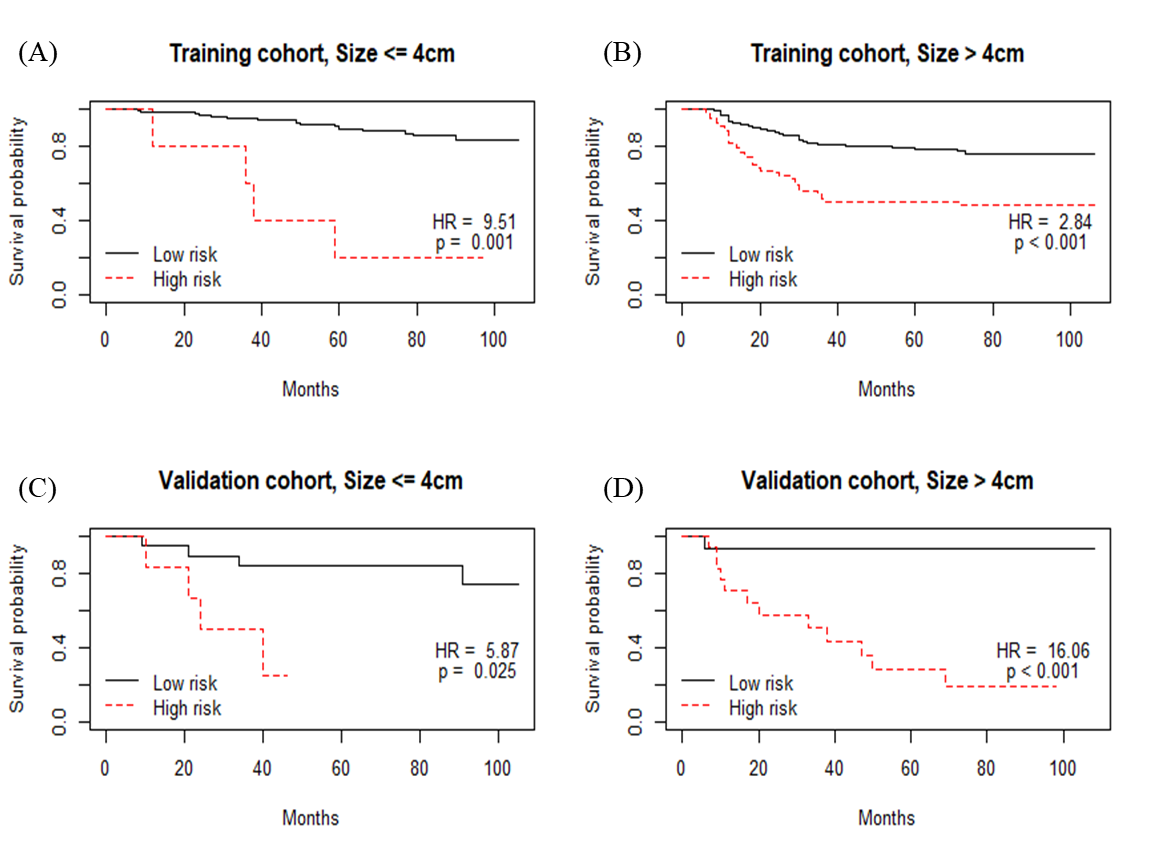
Figure S4. Kaplan-Meier survival analysis of recurrence-free survival according to the radiomics score classifier in subgroups of the training and validation cohorts.** (A) Training cohort, size < 4 cm on CT (n = 136). (B) Training cohort, size ≥ 4 cm on CT (n = 213). (C) Validation cohort, size < 4 cm (n = 27). (D) Validation cohort, size ≥ 4 cm on CT (n = 34). R software (version 3.3.2, <https://www.r-project.org>) was used to draw.

**
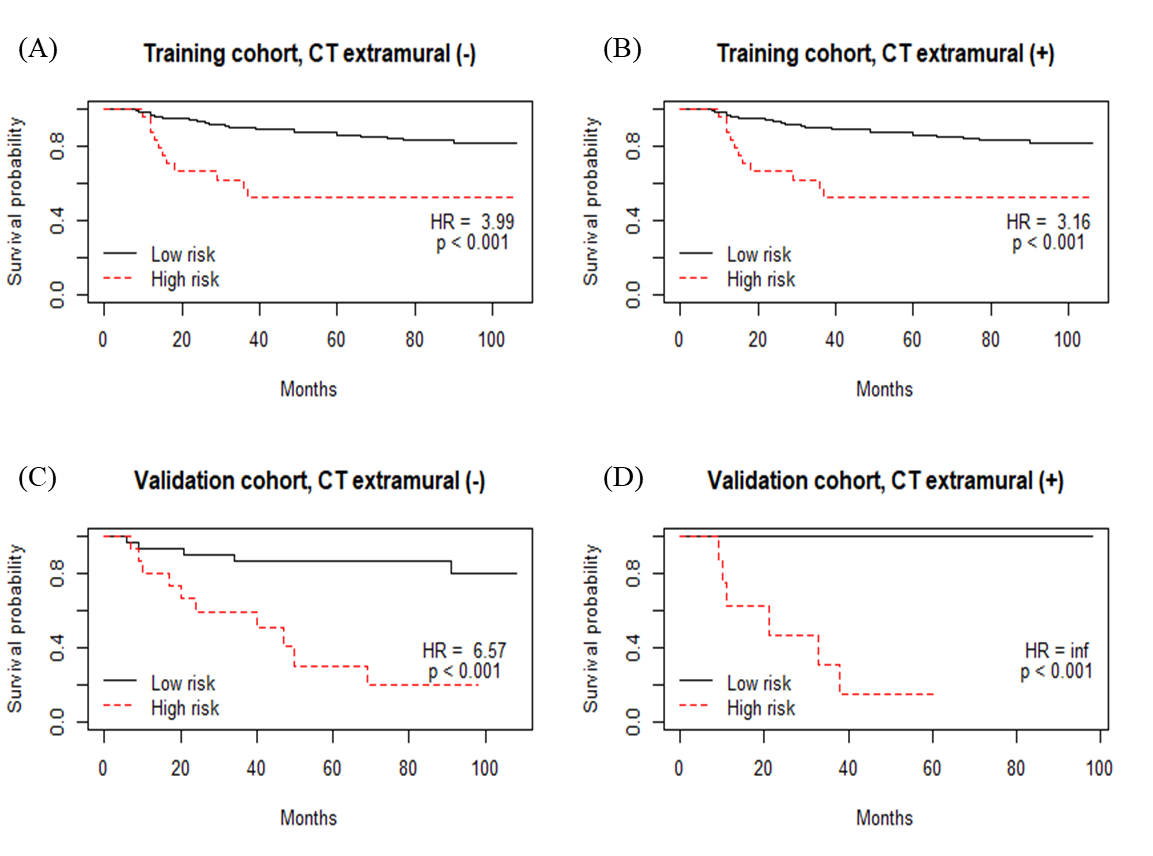
Figure S5. Kaplan-Meier survival analysis of recurrence-free survival according to the radiomics score classifier in subgroups of the training and validation cohorts.** (A) Training cohort, extramural nodular infiltration (-) on computed tomography (CT) (n = 211). (B) Training cohort, extramural nodular infiltration (+) on CT (n = 138). (C) Validation cohort, extramural nodular infiltration (-) (n = 49). (D) Validation cohort, extramural nodular infiltration (+) on CT (n = 12). R software (version 3.3.2, <https://www.r-project.org>) was used to draw.

**
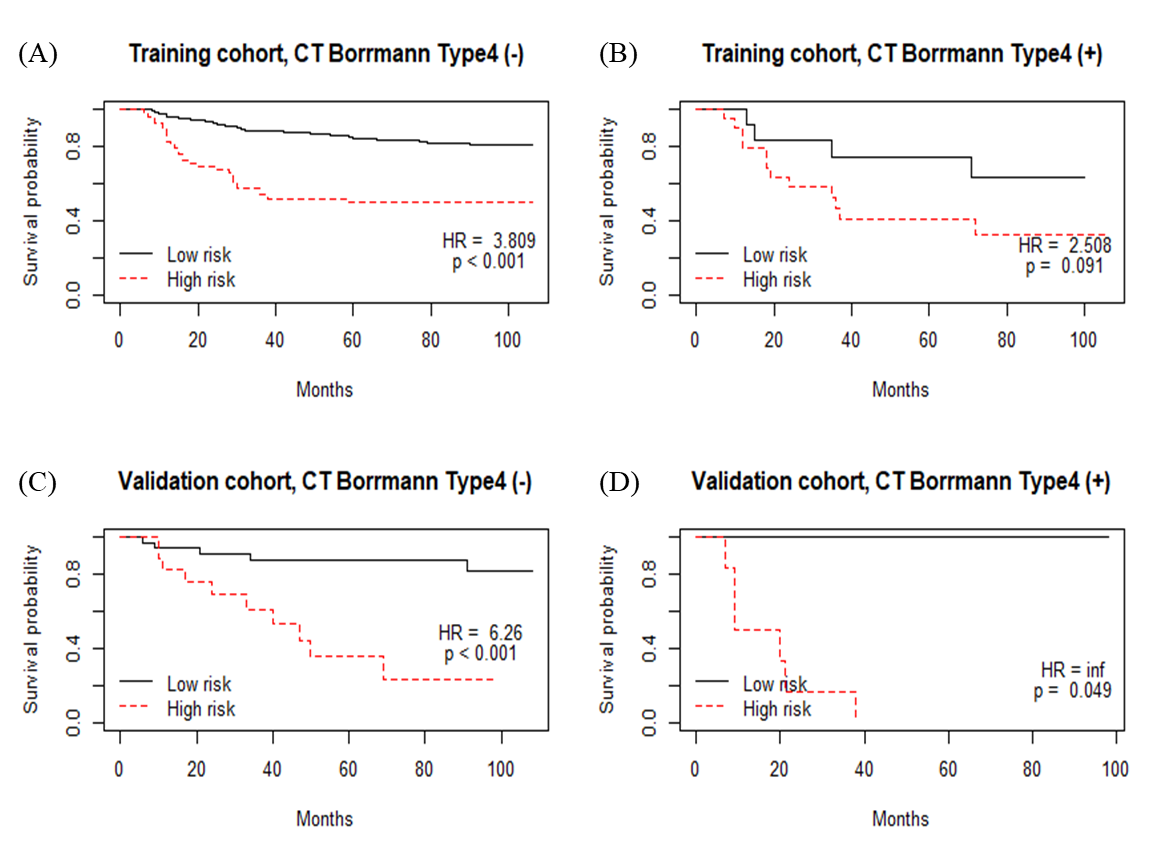
Figure S6. Kaplan-Meier survival analysis of recurrence-free survival according to the radiomics score classifier in subgroups of the training and validation cohorts.** (A) Training cohort, Borrmann type 4 (-) on computed tomography (CT) (n = 318). (B) Training cohort, Borrmann type 4 (+) on CT (n = 31). (C) Validation cohort, Borrmann type 4 (-) (n = 53). (D) Validation cohort, Borrmann type 4 (+) on CT (n = 8). R software (version 3.3.2, <https://www.r-project.org>) was used to draw.

**
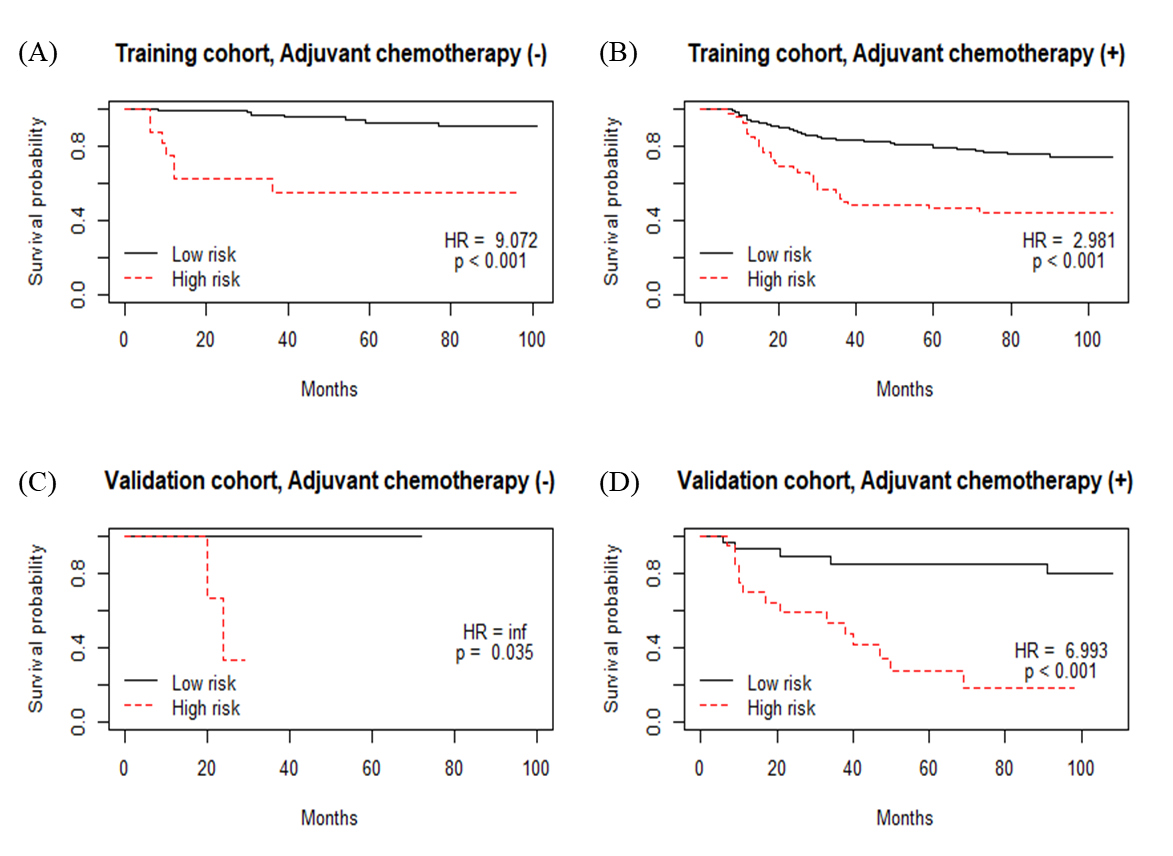
Figure S7. Kaplan-Meier survival analysis of recurrence-free survival according to the radiomics score classifier in subgroups of the training and validation cohorts.** (A) Training cohort, Adjuvant chemotherapy (-) on computed tomography (CT) (n = 110). (B) Training cohort, Adjuvant chemotherapy (+) on CT (n = 239). (C) Validation cohort, Adjuvant chemotherapy (-) (n = 9). (D) Validation cohort, Adjuvant chemotherapy (+) on CT (n = 52). R software (version 3.3.2, <https://www.r-project.org>) was used to draw.


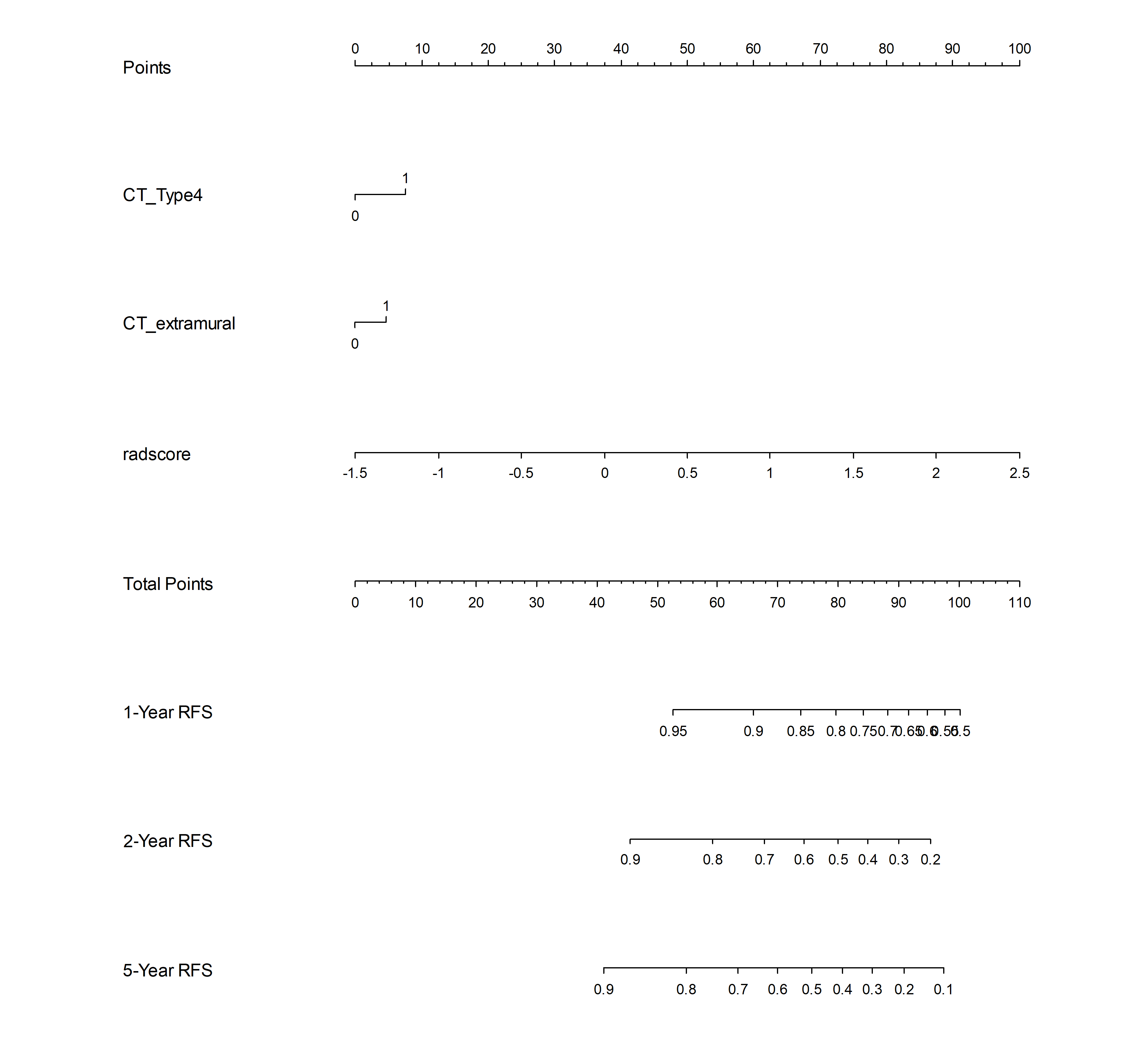


**Figure S8. Merged model Nomogram for recurrence free survival (RFS) at 1, 2 and 5 years.** R software (version 3.3.2, <https://www.r-project.org>) was used to draw.


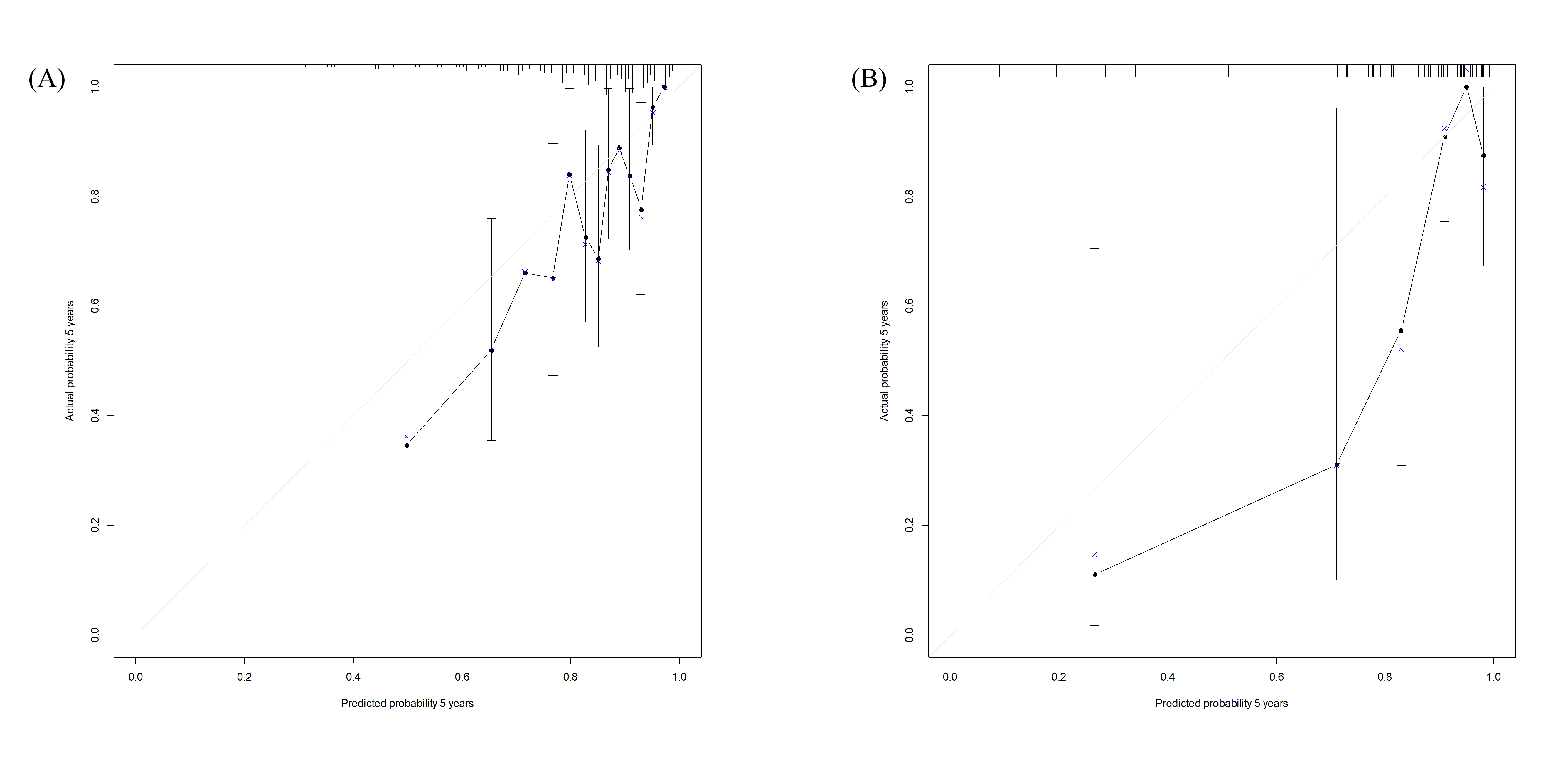


**Figure S9. Calibration curve at 5 years after curative surgery of locally advanced gastric cancer (A) in training cohort and (B) in validation cohort.** R software (version 3.3.2, <https://www.r-project.org>) was used to draw.
